# Uncovering the immune microenvironmental atlas of breast cancer molecular subtypes based on single-cell transcriptomics

**DOI:** 10.1371/journal.pone.0361987

**Published:** 2026-11-06

**Authors:** Shihua Lin, Yangxinyue Zheng, Zushun Chen, Menoudji Djetoyom Patrice, Li Wang, Xia Li, Yunpeng Zhang, Xingda Zhang

**Affiliations:** 1 College of Bioinformatics Science and Technology, Harbin Medical University, Harbin, China; 2 School of Intelligent Medicine and Technology, Big Data Research Center, Hainan Medical University, Haikou, China; 3 Department of Breast Surgery, Harbin Medical University Cancer Hospital, Harbin, China; Michigan Medicine, UNITED STATES OF AMERICA

## Abstract

Breast cancer progression is closely linked to immune cells within the tumor microenvironment (TME), but the immune landscape differences across HER2-positive (HER2+), triple-negative (TNBC), and ER-positive (ER+) subtypes have yet to be systematically characterized. In this study, we characterized immune landscape differences across HER2-positive (HER2+), triple-negative (TNBC), and ER-positive (ER+) breast cancers using single-cell RNA sequencing data, focusing on T cells, macrophages, and B cells. Our analysis revealed that cytotoxic CD8 + T cells were more abundant in TNBC but showed signs of dysfunction. Macrophage subsets, including an NDUFB8 + population, exhibited divergent functional orientations across subtypes. B cells in TNBC exhibited functional alterations, characterized by a decreased proportion of plasmablasts and an increased proportion of memory B cells. Single-cell regulatory network inference and clustering (SCENIC) analysis predicted a set of subtype-specific candidate transcription factors (TFs), including E2F8 across all subtypes and ATF7 in HER2 + tumors, whose activity correlated with clinical outcomes. Finally, mapping multicellular communication networks revealed both conserved and subtype-specific signaling pathways. Together, these results present a detailed transcriptional landscape of immune heterogeneity in breast cancer and contribute novel insights that may inform the design of subtype-specific immunotherapies.

## 1. Introduction

Breast cancer, as one of the most common malignant tumors in women, has been proven that its malignant cells originate from epithelial cells. The occurrence of breast cancer is triggered by genomic alterations in breast epithelial cells and the loss of tissue homeostasis [1]. Meanwhile, breast cancer is a heterogeneous disease shaped by both genetic and environmental factors, and it also exhibits a high degree of intratumoral heterogeneity. Specifically, due to differences in cellular traits, genetics, epigenetics, and molecular expression profiles, various cancer cells within the same tumor exist in distinct metabolic states. These molecular expression patterns are neither uniform nor constant; instead, they evolve dynamically over time as the tumor spreads to different anatomical locations [2]. Clinically, there are different treatment regimens for different subtypes of breast cancer. Currently, breast cancer is mainly clinically stratified based on the expression status of estrogen receptor (ER), progesterone receptor (PR), as well as the overexpression of HER2 or the amplification of the HER2 gene ERBB2. As a result, three major subtypes related to prognosis and applicable for defining treatment strategies have been formed, namely the luminal type (ER + , PR + /-), the HER2-positive (HER2+) type (HER2 + , ER + /-, PR + /-), and triple-negative breast cancer (TNBC; ER − , PR − , HER2−) [3].

The tumor microenvironment (TME), particularly its immune components, plays a critical role in breast cancer progression and therapeutic response. The immune landscape of the TME exhibits substantial heterogeneity across different breast cancer subtypes, characterized by distinct infiltration patterns and functional states [4]. Traditional studies on the TME are confronted with technical limitations: flow cytometry is constrained by spectral overlap, making simultaneous detection of multiple markers difficult; multiplex immunohistochemistry lacks sufficient capability for systematic analysis at the genomic level; and bulk RNA sequencing fails to resolve cellular heterogeneity. The advent of single-cell RNA sequencing technology has overcome these limitations. By revealing the characteristics of individual cells at the whole-transcriptome level, it provides a revolutionary research tool for deciphering cellular heterogeneity and TME complexity in diseases such as breast cancer [5–8]. Single-cell sequencing technology enables high-throughput sequencing analysis of the genome, transcriptome, and epigenome at the single-cell level. It can reveal the gene structure and gene expression dynamics of individual cells and reflect the heterogeneity among cells. Therefore, the pathogenic mechanisms of different subtypes of breast cancer can be better explained from the single-cell level. Single-cell RNA sequencing technology has been applied to better characterize the tumor microenvironment. Previous studies have shown that the breast cancer transcriptome has extensive intratumoral heterogeneity [9]. Thus, understanding the phenotypes of immune cells in the tumor microenvironment is crucial for understanding the mechanisms of cancer progression and responses to immunotherapy. Previous studies have also provided us with a comprehensive understanding of the phenotypic diversity of immune cells in breast cancer [10]. T cells mediate cellular immunity by directly killing target cells, while B cells serve as core effectors of humoral immunity through antibody secretion [11–13]. Macrophages, as the most dominant myeloid lineage cells, exhibit high functional plasticity, including phagocytosis, antigen presentation, and immunomodulation [14]. Therefore, this study focuses on the analysis of T cells, macrophages, and B cells. Existing studies have indicated that tumor-associated macrophages have multiple subtypes and functions, which influence the functions of other immune cells [15]. For example, Yupeng Zhang, Fan Zhong, and others precisely classified tumor-associated macrophages in breast cancer into seven different subtypes through single-cell transcriptome analysis and revealed their multiple roles in tumor immunity [5]. Tiia J Honkanen, Antti Tikkanen and others found in their study on HER2 + metastatic breast cancer that iNOS + M1-like macrophages were significantly associated with improved survival rates [16]. Existing studies have demonstrated that CD8 + T cells in the TME enter a state of functional exhaustion due to the persistent activation of their T cell receptors. This state is characterized by the loss of effector functions and the upregulation of inhibitory receptors, ultimately leading to the failure of anti-tumor immunity [17]. Siyu Guo, Xinkui Liu and others investigated the T-cell-related prognostic risk model and the regulation of the tumor immune microenvironment in TNBC [18]. Sanghoon Lee, Youngbin Cho and others elucidated the interactions between cancer cells and macrophages by analyzing single-cell RNA sequencing (scRNA-seq) data from 25 ER+ tumors [19]. Qing Wang, Ke Sun and others studied and explained how B cells and tertiary lymphoid structures (TLS) significantly promote anti-tumor immunity at both the single-cell and clinical levels. These results will provide more relevant information regarding the applicability and effectiveness of tumor immunotherapy [20].While prior scRNA-seq studies have constructed comprehensive atlases of breast cancer [3,21], they have not systematically compared the functional states of T cells, macrophages, and B cells across subtypes or identified the transcriptional regulators underlying these differences. The present study addresses this gap by integrating immune cell profiling, SCENIC regulatory network analysis to identify subtype-specific candidate transcription factors that may drive immune heterogeneity.

This study aims to systematically dissect the immune microenvironment heterogeneity across HER2 + , TNBC, and ER+ breast cancer subtypes by characterizing the functional states of T cells, macrophages, and B cells. Through integrated analysis, we seek to identify subtype-specific transcriptional regulatory networks and elucidate their role in tumor progression, with a particular focus on transcription factors whose activity correlates with clinical outcomes. By mapping multicellular communication networks, we aim to uncover both conserved and subtype-specific signaling pathways that shape immune cell function, this study seeks to provide a detailed transcriptional atlas of immune heterogeneity in breast cancer and offer novel insights for the development of subtype-specific immunotherapeutic strategies.

## 2. Methods

### 2.1. Data sources

Single-cell RNA sequencing data of the GSE176078 [3] dataset were obtained from the GEO database (https://www.ncbi.nlm.nih.gov/geo/). This dataset was originally generated by Wu et al. (2021) using the 10X Genomics Chromium platform on primary breast tumor specimens obtained from surgical resections of treatment-naïve patients [3]. The dataset comprises samples from 26 patients across three major breast cancer subtypes, including 5 HER2-positive (HER2+), 10 triple-negative (TNBC), and 11 estrogen receptor-positive (ER+) cases. According to the original publication, the cohort included stage I (n = 8), stage II (n = 12), stage III (n = 4), and stage IV (n = 2) breast cancers [3]. After quality control, 93,353 cells were retained for analysis, with 18,504 from HER2 + , 39,593 from TNBC, and 35,256 from ER+ tumors. Bulk RNA-seq data and corresponding clinical information of breast cancer were obtained from The Cancer Genome Atlas (TCGA) database (https://portal.gdc.cancer.gov/). A total of 1,045 patients were included in the survival analysis, including 82 HER2 + samples, 192 ER+ samples, and 771 TNBC samples.

### 2.2. Cell clustering and cell type annotation

Single-cell RNA sequencing data were preprocessed using the Seurat package. Genes expressed in at least three cells and cells expressing at least 200 genes were retained. Cells with more than 20% mitochondrial reads were excluded. After quality control, the data were normalized using the NormalizeData function and scaled with ScaleData. The top 2000 highly variable genes were identified using FindVariableFeatures, followed by principal component analysis (PCA). Clustering was performed using the FindNeighbors and FindClusters functions with a resolution parameter set to 0.8. Cell types were annotated based on a set of classic marker genes. To ensure the accuracy of cell type annotation, we further performed functional enrichment analyses, including Gene Ontology (GO) and Kyoto Encyclopedia of Genes and Genomes (KEGG) pathway analyses, on the differentially expressed genes of each cell type. Differentially expressed genes were identified using the Wilcoxon rank-sum test with an adjusted p-value < 0.05 (Benjamini-Hochberg correction). To assess transcriptional patterns of shared cell types across the three breast cancer subtypes (HER2 + , TNBC, ER+), single-cell expression data were aggregated by cell type within each subtype (pseudobulking). The top 500 highly variable genes shared across all subtypes were identified, and the resulting matrix was z-score normalized across all subtypes. Columns were annotated by subtype and cell type, enabling direct comparison of conserved cell type features and subtype-specific differences. To explicitly account for inter-patient variability and rule out pseudoreplication arising from individual single cells, we performed patient-level pseudobulk analysis focusing on macrophage populations. Raw UMI counts were summed for all cells originating from the same patient, macrophage subset, and tumor molecular subtype. Each aggregated profile corresponds to an independent patient-level pseudobulk sample. This approach was used to validate the marker signature of Macro_NDUFB8 macrophages.

### 2.3. Identification of the biological functions and pathways of specific cell subsets

We employed the clusterProfiler R package to conduct functional enrichment analysis on the differentially expressed genes. P-values were adjusted using the BH method for multiple testing correction, and significantly enriched biological functions and pathways were identified with an adjusted p-value < 0.05. In addition, to further dissect the molecular characteristics of specific cell subsets, we utilized the GSVA (Gene Set Variation Analysis) R package for pathway enrichment analysis. GSVA is a non-parametric and unsupervised gene set enrichment method. It calculates the enrichment scores of specific gene sets based on the gene expression matrix to evaluate the activity of gene sets in samples or cell subsets.In this study, we selected 50 Hallmark gene sets (h.all.v2023.2) as the background gene sets. These gene sets cover pathways related to key biological processes such as cancer development, cell proliferation, immune regulation, metabolic reprogramming, and signal transduction.

### 2.4. Identification of tumor cells in breast cancer subtypes

To identify malignant cell populations within epithelial cells, we used the inferCNV R package to infer copy number variations (CNVs) from single-cell transcriptomic data, employing non-epithelial cells (immune and stromal cells) as normal references. Briefly, genes expressed in fewer than 10 cells or with a mean log2 expression below 0.1 were filtered. Expression values were then converted to Z-scores and winsorized to the range [−3, 3]. Genes were sorted by chromosomal position, and a 100-gene sliding window was used to compute moving averages, with smoothing performed by centering across genes. Cells were classified as malignant based on two metrics: CNV signal strength (MS, mean square), where MS > 0.02, or correlation (CORR) with the top 5% of cells exhibiting the highest CNV signals, where CORR > 0.2.

### 2.5. Identification of candidate transcription factors(TFs) in breast cancer subtypes

In this study, we used the Python-based pySCENIC software package to conduct transcription factor regulatory network analysis, aiming to systematically identify the key regulatory candidate TFs of different cell types. First, we obtained the necessary reference data from public databases, including: (1) A list of human TFs (source:https://raw.githubusercontent.com/aertslab/pySCENIC/master/resources/hs_hgnc_tfs.txt); (2) A motif-TF annotation database (source: https://resources.aertslab.org/cistarget/motif2tf/motifs-v9-nr.hgnc-m0.001-o0.0.tbl);(3) A genome ranking database (source:https://resources.aertslab.org/cistarget/databases/homo_sapiens/hg38/refseq_r80/mc9nr/gene_based/hg38__refseq-r80__10kb_up_and_down_tss.mc9nr.feather). Using the normalized expression matrix generated by the Seurat software as input data, we constructed cell-type-specific transcriptional regulatory networks through the standard analysis pipeline of pySCENIC, which includes GRNBoost2 network inference, cisTarget motif enrichment analysis, and AUCell activity score calculation. This enabled us to systematically analyze the core regulatory TFs of different cell types.

### 2.6. Survival analysis

We downloaded breast cancer RNA-seq data and corresponding clinical information from the TCGA database (TCGA-BRCA project). After excluding patients with missing survival data, follow-up duration shorter than 30 days, and non-primary tumor samples, a total of 1,045 patients were retained for survival analysis, including 82 HER2 + , 771 ER + , and 192 TNBC cases. Molecular subtypes of TCGA-BRCA primary tumor samples were assigned using the PAM50 intrinsic subtyping algorithm [22,23]. In the risk tables below each Kaplan-Meier survival curve, the “Number at risk” indicates the number of patients remaining in each group at each time point (e.g., 0, 50, 100, 150 months), with decreasing numbers over time reflecting patients who were censored due to loss of follow-up or who experienced the event (death or recurrence). For each candidate transcription factor, we determined the optimal cutoff value using the surv_cutpoint function from the survminer R package, which selects the expression threshold that maximizes the log-rank test statistic, and then stratified patients into high-and low-expression groups. The purpose of this survival analysis was to validate the clinical prognostic value of the candidate transcription factors identified in our single-cell RNA sequencing analysis, thereby providing independent data supporting the potential clinical relevance of these transcription factors in breast cancer.

### 2.7. Characterize the interaction between macrophages and T cells based on cell-cell communication analysis

We employed the cellchat R package to evaluate the intercellular interactions between subsets. Cellular communication probabilities were computed using default parameters, and interactions involving cell groups with <10 cells were filtered out. Significance was assessed via permutation testing, with ligand–receptor pairs exhibiting a p-value < 0.05 considered statistically significant. The resulting communication networks were visualized using circle plots and heatmaps to illustrate the communication patterns between tumor and immune cell subsets.

### 2.8. Identification of the tissue tropism of tumor cell subsets

To characterize the tissue distribution of clusters, we calculated odds ratios (ORs) to indicate the tropism. Specifically, for each meta-cluster i and its corresponding cancer subtype j, a 2 × 2 contingency table was constructed. This table contains the number of cells of meta-cluster i in cancer j and in other cancers, as well as the number of cells not belonging to meta-cluster i in cancer j and in other cancers. Then, a Fisher’s exact test was performed on this contingency table to obtain the OR and the corresponding p-value. The p-values were adjusted using the p.adjust function in R with the Benjamini-Hochberg (BH) method. We found that the adjusted p-values for all ORs > 1.5 or ORs < 0.5 were < 1e-10. Therefore, a higher OR value > 1.5 indicates that meta-luster i has a greater tendency to be distributed in cancer j, while a lower OR value<0.5 indicates that meta-cluster i has a lower tendency to be distributed in cancer j.

## 3. Results

### 3.1. Single-cell landscapes of the immune microenvironment in breast cancer subtypes

To systematically analyze the immune microenvironment across HER2 + , TNBC, and ER+ breast cancer subtypes, we conducted clustering analysis on single-cell RNA sequencing data using the Seurat R package. (Fig 1A-C). In addition, to facilitate cross-subtype comparison of gene expression patterns, we generated an integrated heatmap displaying the top 500 most variable genes across shared cell types using the ComplexHeatmap package (S1 Fig). By annotating these cell clusters with a set of classic cell-type marker genes (S2A-B Fig), we found that the immune microenvironment of the three breast cancer subtypes mainly consisted of eight cell types, including B cells, T cells, myeloid cells, endothelial cells, epithelial cells, mesenchymal cells, plasma cells, and proliferating cells (Fig 1 D-F). Further analysis revealed significant differences in the cell-type distribution among the three breast cancer subtypes (S2C Fig). Specifically, T cells were the most abundant immune cell type in all three subtypes. The proportion of T cells was the highest in the HER2 + subtype (Fig 1 J-L), significantly higher than that in the TNBC and ER+ subtypes. The proportion of B cells was relatively low in the TNBC subtype (Fig 1 H). In addition, the proportion of myeloid cells in the TNBC subtype was significantly higher than that in the HER2+ and ER+ subtypes. The high proportion of myeloid cells in TNBC reveals the complexity of its immune microenvironment, which may either promote immune activation through inflammatory responses or exacerbate immune escape through myeloid polarization.Among non-immune cells, the proportion of epithelial cells in the ER+ subtype was significantly higher than that in the HER2+ and TNBC subtypes. The proportions of proliferating cells and plasma cells were low in all subtypes.These results indicate that the immune microenvironments of the HER2 + , TNBC, and ER+ subtypes of breast cancer exhibit significant heterogeneity, which may provide an important theoretical basis for personalized treatment of different breast cancer subtypes.

**Fig 1:**
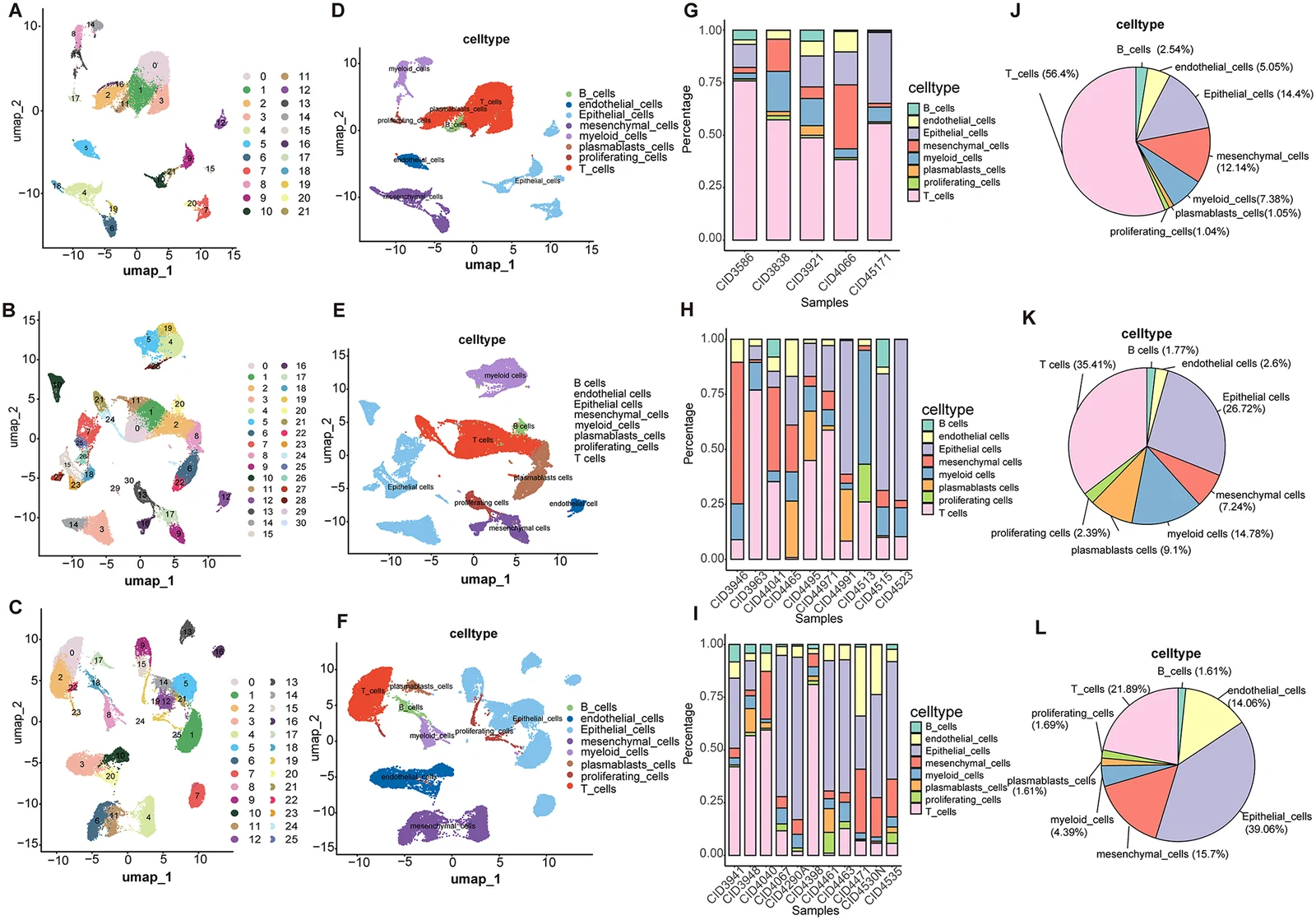
Single-cell landscape of the tumor immune microenvironment in HER2 + , TNBC, and ER+ breast cancer subtypes. (A-C) Cell cluster distribution of the three breast cancer molecular subtypes visualized via UMAP dimensionality reduction: (A) HER2 + subtype, (B) TNBC subtype, (C) ER+ subtype; (D-F) Cell clusters of the three breast cancer subtypes were annotated and grouped using a panel of classical cell type markers, showing the distribution of major cell types in UMAP plots. These major cell types include 8 categories: B cells, T cells, myeloid cells, endothelial cells, epithelial cells, mesenchymal cells, plasma cells, and proliferating cells; (G-I) Bar charts showing the proportion of each cell type among patients for the three subtypes; (J-L) Pie charts representing the proportional analysis of cell types in the three subtypes.

### 3.2. Characterize the functional heterogeneity of tumor cells in different subtypes of breast cancer

To investigate the functional heterogeneity of tumor cells across subtypes, we focused on epithelial cells and applied inferCNV analysis to identify malignant cells in HER2 + , TNBC, and ER+ tumors. Tumor cells were successfully identified in each subtype (Fig 2A). To further characterize functional disparities between malignant and non-malignant cells across three breast cancer subtypes, we identified subtype-specific differentially expressed genes (DEGs) in tumor versus non-tumor cell populations and performed functional enrichment analysis. Distinct DEG profiles were observed among subtypes: HER2+ and TNBC subtypes were dominated by gene upregulation, whereas the ER+ subtype was markedly enriched for downregulated genes. Notably, the number of upregulated genes far exceeded downregulated genes in both HER2+ and TNBC subtypes, while in the ER+ subtype, downregulated genes were approximately fourfold more abundant than upregulated genes (S3A Fig). Venn diagram analysis revealed a 21% overlap in upregulated genes between HER2+ and TNBC subtypes (S3C Fig). Functional enrichment analysis showed that upregulated genes in HER2+ and TNBC subtypes, as well as downregulated genes in the ER+ subtype, were mainly enriched in biological processes including RNA catabolic process, G2/M transition of the mitotic cell cycle, and neutrophil-mediated immunity (S3B Fig). Pathway enrichment analysis indicated these genes were enriched in pathways such as high-rate anabolic metabolism, oxidative phosphorylation, and persistent inflammatory signaling—pathways relatively repressed in ER+ but strongly activated in HER2+ and TNBC subtypes, which underlies the core biological disparities among the three subtypes (S3 Table) [24–26]. These functions are closely associated with the malignant proliferation of cancer cells, which mainly proliferate via mitosis but in an uncontrolled manner, leading to malignant expansion [27]. The RNA catabolic process and G2/M transition of the mitotic cell cycle are key features of cancer cell proliferation [28,29]. Neutrophils, which play a vital role in innate cellular immunity, promote breast cancer progression by facilitating cancer cell proliferation, angiogenesis, metastasis, and establishing an immunosuppressive microenvironment [30]. Additionally, upregulated genes in HER2+ and TNBC subtypes were significantly enriched in p53-like mediator signal transduction; p53 is critical in regulating the tumor immune microenvironment, and its inactivation may serve as a potential target for immunotherapy [31]. These findings reveal the functional heterogeneity of tumor cells among breast cancer subtypes, providing important clues for further exploration of their biological mechanisms and the development of targeted therapeutic strategies.

**Fig 2:**
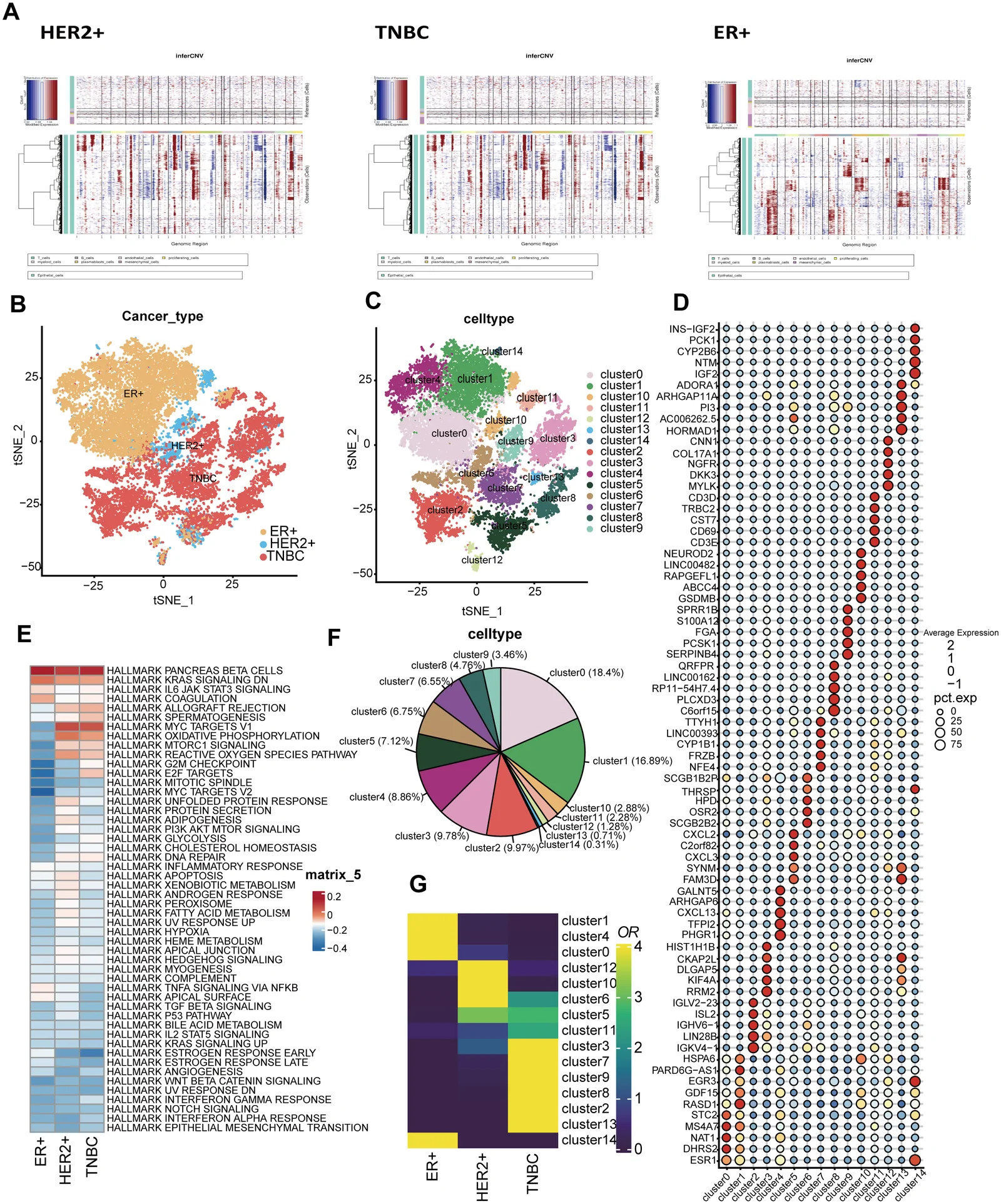
Analysis of tumor cells in three subtypes. (A) Identification of tumour cells in three subtypes of breast cancer epithelial cells; (B-C) Integration of the three subtypes of tumour cells for downscaling visualization; (D) Dot plot demonstrating top5 differential genes for each cell cluster; (E) Gsva analysis of the three subtypes of tumour cells; (F) Pie chart demonstrating the percentage of tumour cells in cell clusters, (G) We calculated odds ratios (OR) to indicate preference, which are used to characterize the tissue distribution of tumor cell clusters.

Clustering analysis of tumor cells across the three breast cancer subtypes identified 15 subclusters (Fig 2B-C), with cluster 0 accounting for the highest proportion (Fig 2F). Tissue tropism analysis showed that cluster 5, 6, 10, and 12 were predominantly composed of HER2 + breast cancer cells; cluster 2, 3, 7, 8, 9, and 13 of TNBC cells; and cluster 0, 1, 4, and 14 of ER+ cells (Fig 2G). Fig 2D presents the top 5 differentially expressed genes in each subcluster. In cluster 0, ESR1 mutation is an emerging clinical biomarker for metastatic ER-positive breast cancer [32]. NAT1, upregulated in luminal breast cancer, promotes bone metastatic niche formation and bone metastasis via the NAT1/NF-κB/IL-1B axis [33]. DHRS2 is considered a potential marker for breast cancer metastasis [34], and STC2, closely linked to the development and metastasis of multiple cancers, may serve as a cancer biomarker [35]. Additionally, reduced STC2 expression is associated with endocrine therapy resistance in ER+ inflammatory breast cancer and advanced disease [36]. GSVA analysis was performed to explore functional differences in tumor cells among the three breast cancer subtypes (Fig 2E). All three subtypes showed significant enrichment in KRAS SIGNALING DN, indicating shared regulatory features of the KRAS signaling pathway across breast cancer subtypes with distinct molecular profiles. This pathway is critically associated with tumor initiation, progression, immune microenvironment, and therapeutic response, consistent with previous studies showing that KRAS signaling-enriched TNBC correlates with a favorable tumor immune microenvironment and improved survival [37]. The ER+ subtype was significantly enriched in the IL6/JAK/STAT3 SIGNALING pathway, which plays a key role in cell proliferation and differentiation.Existing literature has shown that the IL6/JAK/STAT3 pathway promotes the development of endocrine therapy resistance in ER+ breast cancer through crosstalk with estrogen receptor signaling [38]. In HER2 + breast cancer, inhibition of the autocrine IL-6-JAK2-STAT3-calprotectin axis serves as a targeted therapy for HR-/HER2 + breast cancers [39]. In TNBC, Wwox inhibits the metastasis and proliferation of TNBC cells via the JAK2/STAT3 pathway [40].Its activation is closely related to the formation of the tumor inflammatory microenvironment and plays an important role in the occurrence and development of various tumors [41].

Additionally, the HER2+ and TNBC subtypes were mainly enriched in the MYC TARGETS V1 pathway; MYC has been shown to promote TNBC immunosuppression by inhibiting interferon signal transduction [42]. These results not only reveal the molecular pathway-level heterogeneity of tumor cells across different breast cancer subtypes but also clarify their potential associations with the tumor immune microenvironment and clinical prognosis. The positive role of KRAS signaling in TNBC, the pro-inflammatory effect of the IL6/JAK/STAT3 pathway in the ER+ subtype, and the immunosuppressive effect of MYC signaling in HER2+ and TNBC subtypes provide important theoretical bases for developing subtype-specific targeted and immunotherapeutic strategies.

### 3.3. Macro_NDUFB8 exhibit dynamic functional switching and immune evasion mechanisms across different breast cancer subtypes

To analyze myeloid cell heterogeneity across HER2 + , TNBC, and ER+ subtypes, we performed dimensionality reduction and clustering analysis on myeloid cells from each subtype. Results showed that the HER2 + , TNBC, and ER+ subtypes were clustered into 9, 23, and 12 subclusters, respectively. Each subcluster was annotated and named based on the expression of a set of classic marker genes: the HER2+ and ER+ subtypes were each divided into 5 subcategories (cDC1, cDC2, pDC, macrophages, and monocytes; Fig 3A, 3C), while the TNBC subtype was divided into 6 subcategories (cDC1, cDC2, DC, pDC, macrophages, and monocytes; Fig 3B). As shown in the bar and pie charts in Fig 3A-C, macrophages accounted for the highest proportion across all three subtypes, and monocytes also constituted a relatively large proportion in the TNBC and ER+ subtypes. In contrast, dendritic cells (cDC1, cDC2, DC, pDC) were less abundant in the HER2+ and ER+ subtypes and even absent in the TNBC subtype. Broz et al. demonstrated that cDC1 is the only myeloid subset in the tumor microenvironment capable of efficiently cross-presenting antigens and activating naive CD8 ⁺ T cells, with its abundance positively correlated with T cell infiltration and function; genetic depletion of cDC1 completely abrogates anti-tumor CD8 ⁺ T cell immunity, highlighting its critical role as a bridge between innate and adaptive immunity [43]. As the most malignant breast cancer subtype, TNBC disrupts both the local tumor microenvironment and systemic immune system, mainly via impaired dendritic cell function. This impairment prevents dendritic cells from recognizing mutated genes and cancer cells, as well as transmitting tumor antigen information to T lymphocytes, thereby failing to activate anti-tumor specific immune responses and ultimately promoting tumor growth and metastasis [44].

**Fig 3:**
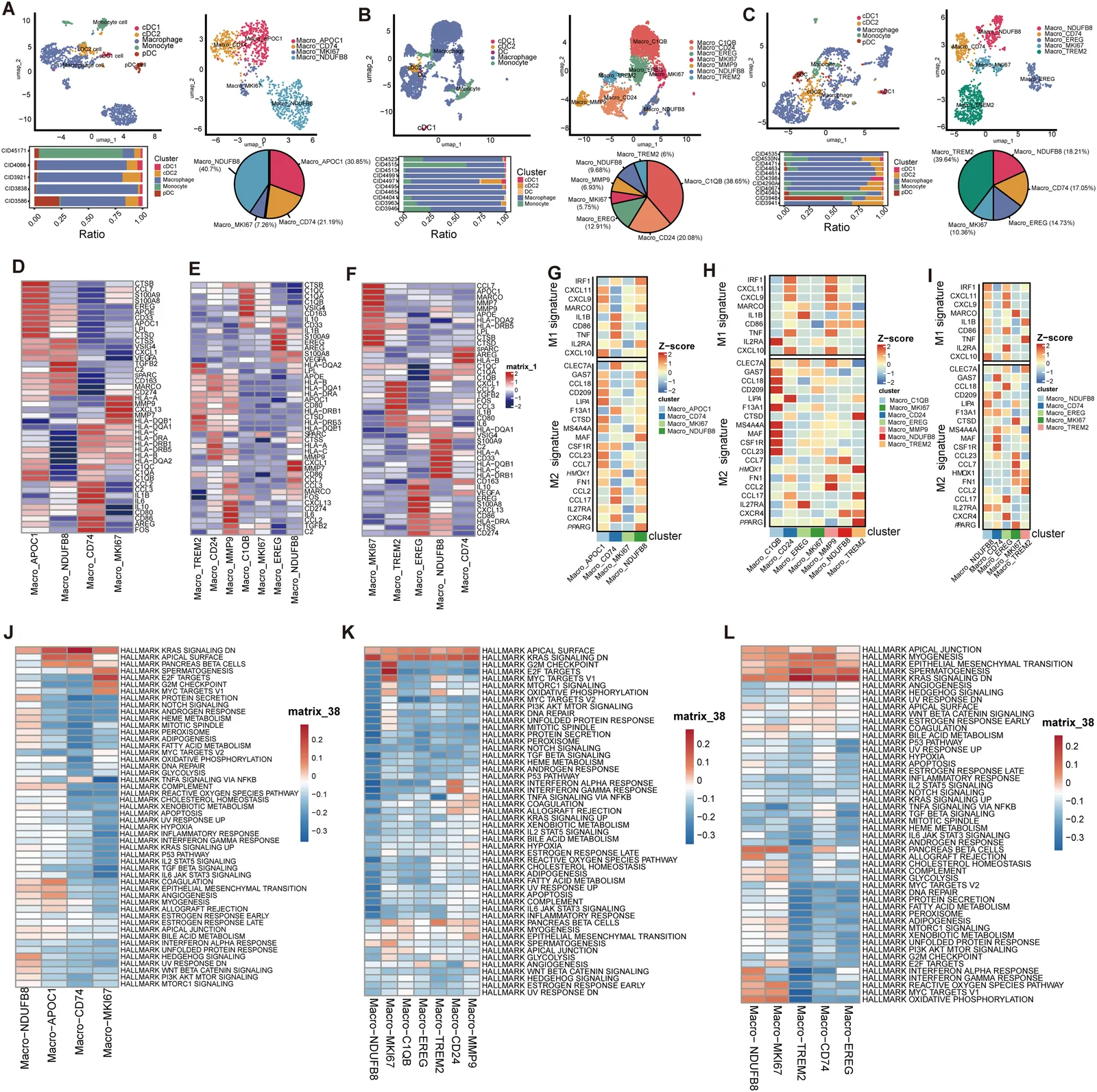
Functional polarization diversity of macrophage subsets and their regulatory mechanisms within the tumor microenvironment. (A) The upper-left Fig shows the UMAP plot of HER2 + myeloid cell subsets. The lower-left part shows the cell proportion components of myeloid cell subsets. The upper-right is the macrophage subset, and the lower-right shows the proportion of cells in the macrophage subset.(B) The figure represents TNBC.(C) The figure represents the ER+ subtype.(D), (E), (F) Respectively show the expression of macrophage-related functional genes in the macrophage subsets of HER2 + , TNBC, and ER+ subtypes.(G), (H), (I) Respectively show the expression of M1 and M2 characteristic genes in the macrophage subsets of HER2 + , TNBC, and ER+ subtypes.(J), (K), (L) Respectively represent the functional analysis of macrophage subsets of HER2 + , TNBC, and ER+ subtypes by GSVA analysis.

Macrophages accounted for the highest proportion among myeloid cells across all three breast cancer subtypes, with high expression of macrophage-associated markers including CXCL10, CXCL11, and CCL18 in HER2+ and ER+ tumors, and CCL17 in TNBC tumors (Fig 3A-C). These results indicate that macrophages play a dominant role among myeloid cells. Based on tumor progression-related and anti-tumor functional gene signatures, HER2 + macrophages were classified into one pro-tumor subset (Macro_NDUFB8), two anti-tumor subsets (Macro_CD74 and Macro_MKI67), and one dual-function subset (Macro_APOC1) (Fig 3D)(S4 Table). Macro_NDUFB8 highly expressed immunosuppressive genes (CD274, CCL2, IL10, TGFB2) promoting tumor progression [45,46]; Macro_APOC1 co-expressed lipid metabolism and complement genes (APOC1, APOE, LPL, C1QA/B/C) alongside pro-angiogenic factors (EREG, VEGFA, AREG), reflecting dual tumor-clearance and pro-tumor activities [47–50]; and Macro_CD74 and Macro_MKI67 exhibited high expression of MHC class I/II genes, costimulatory molecules (CD80, CD86), and pro-inflammatory cytokines (IL1B, IL6), indicating strong antigen-presenting capacity and anti-tumor function, with Macro_MKI67 additionally enriched in proliferation pathways (MYC targets, G2/M checkpoint) (Fig 3J) [51–54]. The Macro_MKI67 subpopulation similarly displayed high expression of MHC class I and class II genes, demonstrating robust antigen-presenting ability, and was significantly enriched in cell proliferation-related pathways such as MYC targets and the G2/M checkpoint as revealed by GSVA analysis (Fig 3J). Notably, Macro_NDUFB8, Macro_APOC1, and Macro_CD74 simultaneously expressed both M1 and M2 macrophage marker genes (Fig 3G), indicating that these subsets may exhibit mixed polarization features rather than fitting strictly into the traditional M1/M2 classification. This observation hints at the potential functional complexity and plasticity of macrophages within the tumor microenvironment [52].

In TNBC, macrophages were classified into five pro-tumor subsets (Macro_C1QB, Macro_EREG, Macro_TREM2, Macro_NDUFB8, Macro_MKI67), one anti-tumor subset (Macro_CD24), and two dual-function subsets (Macro_MMP9) (Fig 3E). The pro-tumor subsets operated through distinct mechanisms: Macro_C1QB via complement activation; Macro_EREG via pro-angiogenic signals; Macro_TREM2 via M2 polarization with MHC expression; Macro_NDUFB8 via inflammatory chemokines (S100A8, CXCL1) and angiogenesis (VEGFA) [53]; and Macro_MKI67 via cell cycle enrichment. In contrast, anti-tumor Macro_CD24 displayed M1-like, antigen-presenting features with high MHC class I/II expression (Fig 3H), while dual-function Macro_MMP9 co-expressed M1 markers, immunosuppressive mediators (CD274, CCL2), and costimulatory molecules (CD80) (Fig 3E). In the ER+ subtype, macrophages were classified into two pro-tumor subsets (Macro_CD74 and Macro_MKI67), one anti-tumor subset (Macro_NDUFB8), and two dual-function subsets (Macro_TREM2 and Macro_EREG) (Fig 3F). Macro_TREM2 expressed immunosuppressive genes (TGFB, CCL2) alongside CD80, reflecting dual functions [54]; Macro_NDUFB8 showed strong antigen-presenting capacity via MHC class I/II; Macro_CD74 expressed pro-angiogenic genes (AREG, VEGFA) and the M2 marker CD163 [55]. Macro_EREG displayed dual functions, co-expressing the immune costimulatory gene CD86 along with angiogenesis-related genes (EREG, VEGFA) and CD163 (Fig 3I) [50]. Macro_MKI67 showed high expression of MHC class II genes (HLA-DQA2, HLA-DRB5), lipid metabolism-related genes (APOE, LPL), and M2-associated markers (CCL7, CTSD), leading to its classification as pro-tumor. These results indicate that ER+ macrophages are highly heterogeneous, and their functional diversity may significantly influence the tumor immune microenvironment. Across the three breast cancer subtypes, all contained cell cycle-related macrophage subclusters, with Macro_NDUFB8 present in each. Notably, Macro_NDUFB8 exhibited pro-tumor functions in HER2+ and TNBC but anti-tumor functions in ER + , suggesting that the tumor microenvironment critically shapes macrophage functional polarization. GSVA analysis revealed that macrophage subclusters from all three subtypes were significantly enriched in the KRAS SIGNALING DN pathway (Fig 3J-L), which has been implicated in driving tumor evasion of innate immune surveillance [56]. Macro_NDUFB8 macrophages in the TNBC subtype showed particularly strong enrichment in this pathway, suggesting their potential involvement in KRAS-mediated immune regulation. To characterize the differentiation states of macrophage subsets across breast cancer subtypes, we performed CytoTRACE2 analysis (S4 Fig). The results revealed a continuous differentiation trajectory in all three subtypes, with subsets distributed along a spectrum from high potency (less differentiated) to low potency (terminally differentiated). Notably, dual-function subsets (Macro_APOC1 in HER2 + , Macro_MMP9 in TNBC, and Macro_TREM2/EREG in ER+) consistently occupied intermediate positions along the trajectories, whereas terminally differentiated pro-tumor (e.g., Macro_NDUFB8 and Macro_C1QB in TNBC) or anti-tumor subsets (e.g., Macro_NDUFB8 in ER+) localized to the low-potency endpoints. These findings demonstrate that macrophage polarization in breast cancer occurs along a continuous differentiation trajectory, and more importantly, that the same macrophage subset, such as Macro_NDUFB8, can acquire opposing functional fates, pro-tumor in TNBC versus anti-tumor in ER + , depending on the specific cues of the subtype-specific tumor microenvironment (S5 Fig)(S6 Fig)(S7 Fig).

To dissect the transcriptional regulatory mechanisms underlying macrophage functional heterogeneity, we applied SCENIC analysis to predict the top 10 most active candidate transcription factors (TFs) for each macrophage subset in HER2 + , TNBC, and ER+ breast cancers (Fig 4A-C). Several candidate TFs were identified across multiple subtypes. HOXB7 and E2F8 were present in all three subtypes (Fig 4E, S1 Table), suggesting they may serve as fundamental regulators in breast cancer, consistent with previous reports on their roles in cell proliferation, apoptosis, and metastasis [57–59]. In the HER2+ and TNBC subtypes, we also identified RAD21, STAT5A, and E2F1. In the HER2+ and ER+ subtypes, we found two pro-tumor-associated candidate TFs (NR1H3 and ERG) and one anti-tumor-associated TF (LHX2). NR1H3 is known to regulate lipid homeostasis and inflammation, while ERG plays roles in proliferation, differentiation, angiogenesis, and apoptosis. The presence of LHX2 in anti-tumor macrophage subpopulations of both HER2+ and ER+ subtypes suggests that LHX2 may enhance macrophage phagocytosis and immune receptor expression, thereby contributing to anti-tumor activity. In the TNBC and ER+ subtypes, we identified MAF, ETV7, HOXB2, VDR, SNAI1, ELF3, and ETS2. Among these, MAF and ETS2 are pro-tumor candidate TFs predominantly enriched in pro-tumor macrophage subsets. MAF may promote cell cycle progression and proliferation, while ETS2 may upregulate matrix metalloproteinases to facilitate tumor invasion and metastasis.

**Fig 4:**
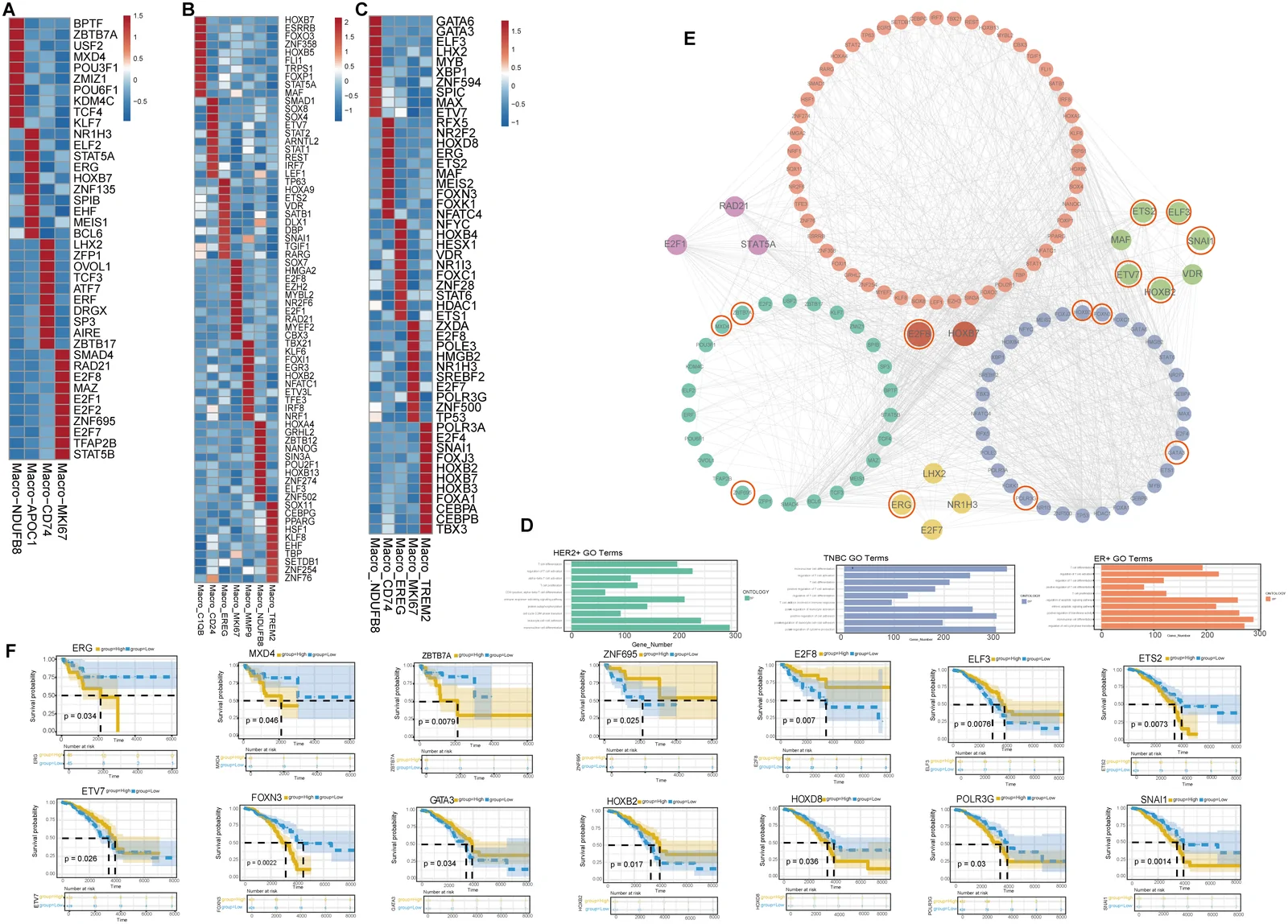
Identification of key candidate TFs in macrophages. (A-C) Transcription factor activity profiles specific to macrophage subsets: Heatmaps of TF activity calculated via the SCENIC method, showing the top 10 most significantly active TFs in each macrophage subset across the HER2+ (A), TNBC (B), and ER+ (C) subtypes; (D) Functional enrichment analysis of TF target genes: GO functional enrichment analysis was performed on the target gene sets regulated by key candidate TFs in the three subtypes. Bar charts display significantly enriched biological processes, with colors representing different functional categories; (E) Construction of a cross-subtype candidate TF regulatory network: A core regulatory network integrated from key candidate TFs of the three subtypes. The size of nodes represents the degree of TFs; (F) Survival analysis of prognosis-related candidate TFs: Based on survival analysis results from the TCGA database, TFs significantly associated with patients’ overall survival (p < 0.05) in the HER2 + , TNBC, and ER+ subtypes are shown.

In addition, we identified several prognosis-related marker genes in HER2 + breast cancer, including ERG, ZBTB7A, and ZNF695, with ERG also associated with ER+ prognosis. ZBTB7A, a tumor suppressor, may promote epithelial-mesenchymal transition (EMT) via NF-κB signaling, thereby enhancing tumor migration, invasion, and metastasis [60,61], while ZNF695 is a zinc finger protein involved in transcriptional regulation and differentiation. ETV7 (a tumor suppressor) and GATA3 (a CD4 + Th2 cell marker) are anti-tumor transcription factors. Notably, STAT6, another Th2 cell marker, was detected in the anti-tumor macrophage subset Macro_EREG in ER+ tumors, suggesting potential crosstalk between anti-tumor macrophages and CD4 + Th2 cells to jointly suppress tumor progression.

By constructing the interaction network between transcription factors and target genes, we extracted the candidate TF interaction subnetworks of the three subtypes (Fig 4E) and observed close interactions among key candidate TFs across different breast cancer subtypes. GO functional analysis revealed that the target genes of these candidate TFs were significantly enriched in multiple T cell-related functions (Fig 4D), suggesting potential crosstalk between macrophages and T cells within the breast cancer immune microenvironment. Furthermore, we identified several candidate TFs associated with patient prognosis: ERG, MXD4, ZBTB7A, and ZNF695 in HER2 + breast cancer; E2F8 in TNBC; and ELF3, ETS2, ETV7, FOXN3, GATA3, HOXB2, HOXB3, POLR3G, and SNAI1 in ER+ breast cancer (Fig 4F).

### 3.4. Cytotoxic CD8 + T cell dysfunction and immune escape mechanisms in TNBC

Based on the close association between the aforementioned macrophage regulatory networks and T cell functions, this study further systematically dissected the composition profile and functional status of T cell subsets. We performed unsupervised clustering on T cells from HER2 + , TNBC, and ER+ tumors and identified CD4 + T cells, CD8 + T cells, NK cells, and cycling cells (Fig 5B). Based on marker gene expression, CD4 + T cells were annotated as naïve (CD4_ANXA1, CD4_CCR7, CD4_TCF7; TCF7 + , SELL + , CCR7+), regulatory Treg (CD4_FOXP3, CD4_TIGIT, CD4_CTLA4; FOXP3 + , CTLA4+), and cytotoxic CD4 + T cells (CD4_PDCD1, CD4_GZMA; GZMA + , GZMK+). CD8 + T cells were annotated as tissue-resident memory TRM (CD8_CD69; CD69+) and cytotoxic CTL (CD8_GZMA, CD8_NKG7, CD8_GZMH, CD8_GZMK, CD8_IFNG, CD8_MKI67, CD8_ANXA1, CD8_CD24, CD8_PFN1, CD8_GZMB; GZMA + , GZMB + , NKG7 + , IFNG+). NK cells were identified as NK_TYROBP (TYROBP + , NKG7+), and cycling cells as Cycling_JCHAIN and Cycling_MS4A1 (MKI67 + , TOP2A+). These annotation patterns were consistent across HER2 + , TNBC, and ER+ subtypes (Fig 5B).

**Fig 5:**
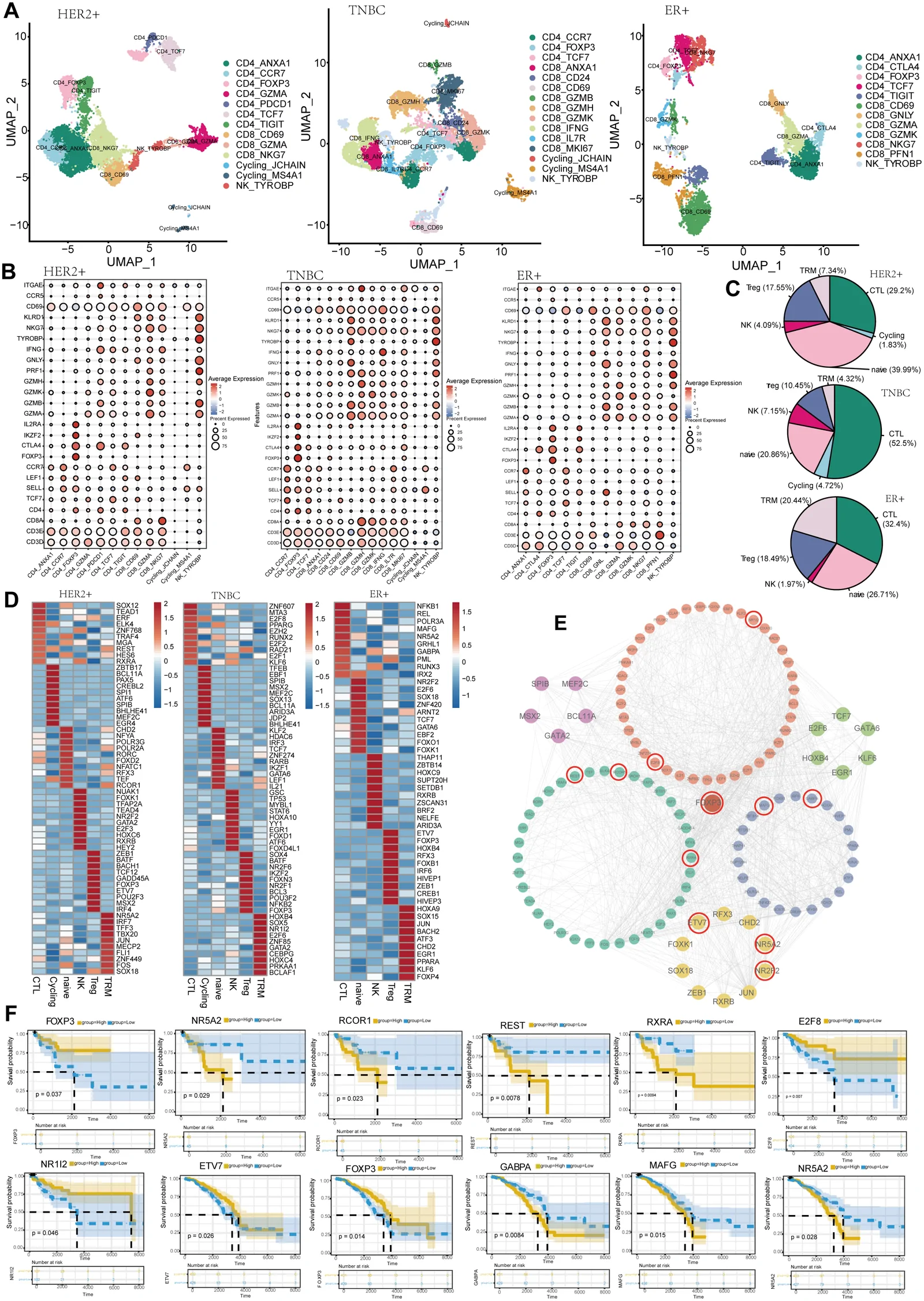
Analysis of T cells in three subtypes of breast cancer. (A) From left to right are the UMAP plots of T-cell subsets in HER2 + , TNBC, and ER+ breast cancers. (B) From left to right are the expression patterns of functional genes in T-cell subsets of HER2 + , TNBC, and ER+ breast cancers.(C) From top to bottom are the proportions of T-cell subsets in HER2 + , TNBC, and ER+ breast cancers.(D) The SECNIC method was used to identify the 10 most significantly active transcription factors in each subset of the HER2 + , TNBC, and ER+ subtypes.(E) The interaction network of key candidate TFs among T cells of the three subtypes.(F) The survival R package was used to analyze the key TFs of the three subtypes. The figure shows the key TFs with significant P-values for survival prognosis.

Cell proportion analysis revealed that TNBC had a significantly higher proportion of cytotoxicity-related cells and a lower proportion of Treg cells compared to HER2+ and ER+ subtypes (Fig 5C). Although CD8 + T cells were abundant in TNBC, the most aggressive subtype, their anti-tumor function may be compromised. Previous studies have shown that tumor cells can evade immune attack by upregulating immune checkpoint molecules such as PD-1/PD-L1 [62]. and can suppress CD8 + T cell activity through immunosuppressive molecules including NOS2 and COX2 [63]. Inhibition of COX2 has been shown to reduce TNBC tumor growth and increase intratumoral CD4+ and CD8 + T cell infiltration [63]. Additionally, the PD-1/PD-L1 pathway plays a key role in maintaining immunosuppression in the TME, and blocking this axis has emerged as a promising treatment strategy for TNBC [64]. Additionally, the PD-1/PD-L1 pathway plays a key role in maintaining immunosuppression in the TME, and blocking this axis has emerged as a promising treatment strategy for TNBC [64]. Collectively, these factors, along with the immunosuppressive tumor microenvironment (including MDSCs, TAMs, Tregs, and cytokines such as IL-10 and TGF-β), may contribute to CD8 + T cell dysfunction in TNBC [65]. Our analysis revealed that cytotoxic CD8 ⁺ T cells in the TNBC subtype were characterized by high enrichment of gene sets related to T cell exhaustion and functional impairment. Meanwhile, these cells also displayed elevated activity of immunosuppressive gene programs, indicating a dysfunctional and immunosuppressive state within the TNBC immune microenvironment(S8 Fig).

To dissect the transcriptional regulatory mechanisms underlying T cell functional heterogeneity, we applied SCENIC analysis to predict candidate TFs in T cells across the three breast cancer subtypes (Fig 5D). In HER2+ and TNBC, we identified five candidate TFs (BCL11A, GATA2, MSX2, SPIB, MEF2C); BCL11A expression was associated with TNBC cell proliferation [66], and MSX2 with breast cancer prognosis [67](Fig 5E). In HER2+ and ER + , we identified ten candidate TFs (ZEB1, SOX18, ETV7, CHD2, JUN, RXRB, NR5A2, FOXK1, RFX3, NR2F2); NR5A2 was associated with prognosis in both subtypes and has been linked to decreased breast cancer risk [68], ETV7 (a tumor suppressor) with ER+ prognosis, and NR2F2 with insulin-induced EMT [69] (Fig 5F). In TNBC and ER + , we identified seven candidate TFs (KLF6, ARID3A, TCF7, GATA6, EGR1, HOXB4, E2F6); KLF6 inhibits estrogen receptor-mediated cell growth in breast cancer via a c-Src-mediated pathway [70], GATA6 is overexpressed in breast cancer and promotes breast cancer cell epithelial-mesenchymal transition by upregulating slug expression [71], and EGR1 exhibits dual tumor-suppressive and oncogenic activities [72]. FOXP3, a Treg marker, was identified in all three subtypes and associated with HER2+ and ER+ prognosis. Additional prognosis-associated candidate TFs included ATF6, BATF, RCOR1, REST, RXRA for HER2 + ; E2F8 and NR1I2 for TNBC; and EBF2, ETV7, GABPA, GRHL1, IRX2, HOXC9, MAFG, NR5A2 for ER+ (Fig 5F). These findings demonstrate heterogeneity in key candidate TFs across breast cancer subtypes and their association with tumor progression and patient prognosis.

### 3.5. Dysregulated B cell differentiation reveals key features of the immunosuppressive microenvironment in TNBC

Given that the subset characteristics and functions of T cells and myeloid cells have been systematically characterized, to comprehensively dissect the immune cell landscape in the TME, we further focused on the B cell population for in-depth analysis. We analyzed single-cell B cell data for each subtype and identified multiple subclusters, which were annotated into three functional populations, namely naïve B cells, memory B cells, and plasmablast B cells, based on classic marker gene expression (Fig 6A). TNBC exhibited a relatively lower proportion of plasmablast B cells and a higher proportion of memory B cells compared to HER2+ and ER+ subtypes (Fig 6B). Given the immunosuppressive nature of the TNBC microenvironment, this imbalance may reflect B-cell state alterations, potentially associated with poor prognosis and reduced immunotherapy response [73,74]. GSVA pathway analysis revealed that B cells across the three subtypes showed enrichment in the KRAS SIGNALING DN pathway, which has been linked to tumor immune escape. Notably, B cells from TNBC tended to display stronger enrichment in the epithelial-mesenchymal transition (EMT) pathway, a biological process relevant to tumor invasion and metastasis and potentially involved in TNBC progression [75] (Fig 6C). Transcriptomic similarity analysis revealed significant differences in the differentiation states of B cells among different subtypes. The naïve B cells and plasma cells of the HER2+ and TNBC subtypes showed a strong transcriptomic similarity, while this similarity was weaker in the ER+ subtype (Fig 6D). This difference may be driven by the heterogeneity of the tumor microenvironment and has an important impact on the immune response and treatment response.

**Fig 6:**
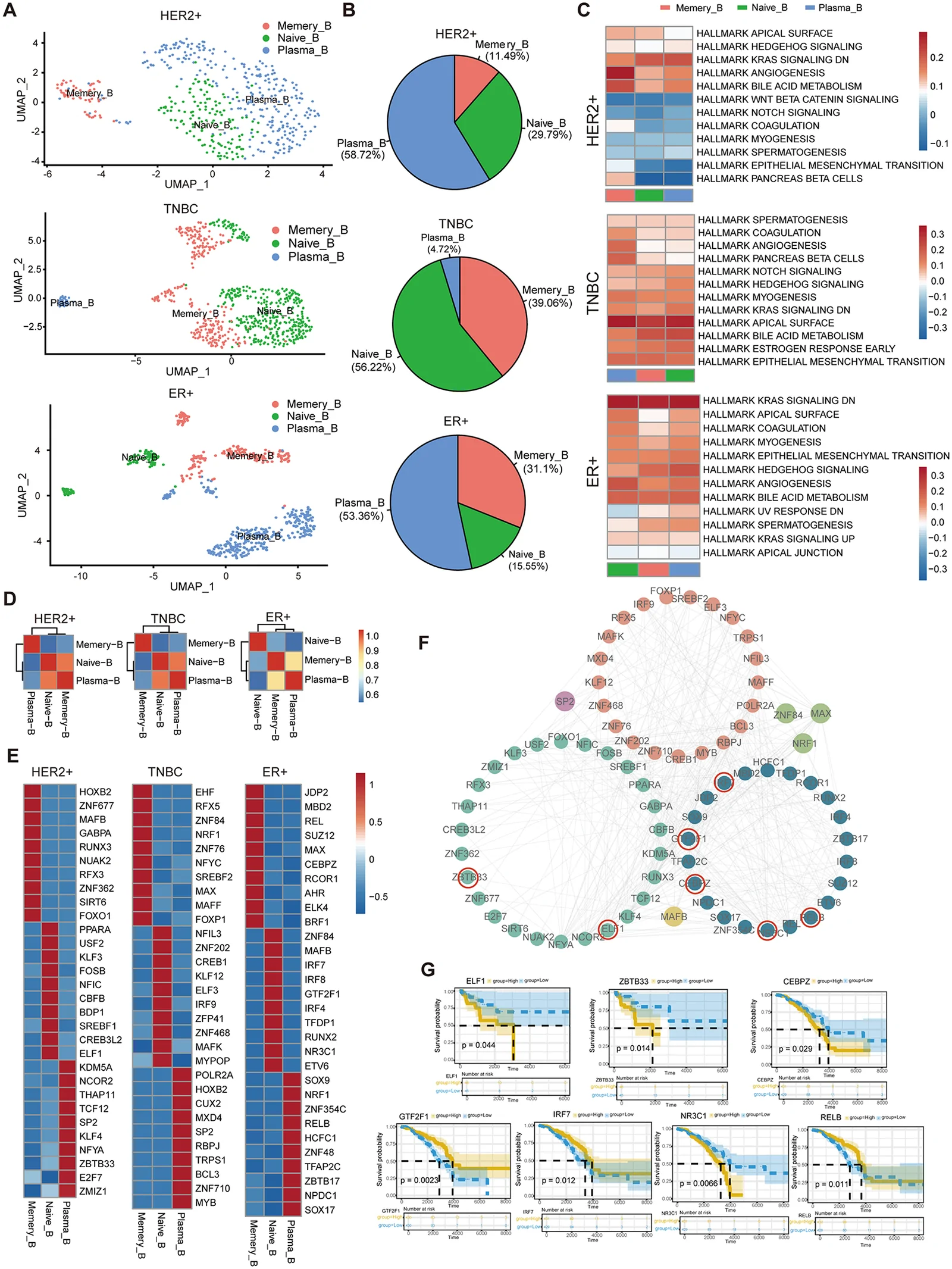
Analysis of B cells in breast cancer of three subtypes. (A) UMAP plots of B cells in HER2 + , TNBC, and ER+ breast cancer, respectively; (B) Proportions of B cell subsets in HER2 + , TNBC, and ER+ breast cancer from top to bottom, respectively; (C) Functional analysis of B cell subsets in HER2 + , TNBC, and ER+ breast cancer from top to bottom, respectively; (D) Similarity analysis of B cell subsets among HER2 + , TNBC, and ER+ breast cancer; (E) Key candidate TFs of B cell subsets in the three breast cancer subtypes; (F) Key TFs interaction network between T cell subsets among the three breast cancer subtypes; (G) Survival analysis of key TFs in the three subtypes was performed using the Survival R package, and the figure shows the key candidate TFs with significant prognostic P-values.

Through SCENIC analysis, we predicted candidate TFs of B cells in each subtype (Fig 6E). TF interaction network analysis revealed both common and subtype-specific regulatory characteristics. SP2 was co expressed in HER2+ and TNBC; previous studies have shown that SP2, a downstream effector of the PI3K/AKT pathway activated by CD93 and integrin β1, promotes tumor proliferation and vasculogenic mimicry in breast cancer, suggesting that targeting SP2 or its upstream regulators may have therapeutic potential [76]. ZNF84, NRF1, and MAX were co expressed in TNBC and ER+ (Fig 6F), and the NRF1 proteasome axis has been identified as a potential target for breast cancer treatment [77]. Survival analysis revealed that ELF1 and ZBTB33 were associated with HER2 + prognosis, while CEBPZ, GTF2F1, IRF7, NR3C1, and RELB were associated with ER+ prognosis (Fig 6G). Collectively, these findings reveal B cell subset heterogeneity across breast cancer subtypes, identify B cell imbalance and subtype specific pathway enrichment in TNBC, and uncover key regulatory and prognosis related molecules in each subtype.The results of survival analysis showed that ELF1 and ZBTB33 were related to the prognosis of the HER2 + subtype; CEBPZ, GTF2F1, IRF7, NR3C1, and RELB were significantly related to the prognosis of the ER+ subtype (Fig 6G). These findings provide new insights into understanding the immune characteristics and prognostic influencing factors of different breast cancer subtypes.

### 3.6. Characterizing tumor heterogeneity based on the interaction between macrophages and T Cells in breast cancer subtypes

To investigate intercellular communication between macrophages and T cells, we performed CellChat analysis and identified significant ligand receptor interactions across subtypes. In HER2 + breast cancer, CD4_ANXA1 was predicted to interact with multiple cell types, and CD4_PDCD1 showed significant interactions with macrophage subsets including Macro_NDUFB8, Macro_MKI67, Macro_APOC1, and Macro_CD74, primarily mediated by MHC-II signaling pathways such as HLA-DQA1-CD4, HLA-DPB1-CD4, and HLA-DPA1-CD4 (Fig 7A) [77]. Therefore, in HER2 + breast cancer, CD4_PDCD1 may inhibit tumor progression through MHC-II mediated interactions. In TNBC, Macro_C1QB and CD8_IFNG exhibited extensive interactions with multiple cell types, with higher macrophage T cell interaction strength than in HER2 + tumors, suggesting more complex immune crosstalk in the TNBC microenvironment. A PTPRC-MRC1 interaction was detected between macrophages and T cells in TNBC; PTPRC modulates CD8 + T cell infiltration to regulate tumor immunity [78], while MRC1, a mannose receptor identifying M2 polarized macrophages, may influence tumor growth [79]. PTPRC and MRC1 may jointly regulate immune infiltration, polarization, and tumor progression in TNBC (Fig 7B). In the ER+ subtype, CD4_ANXA1 and CD8_CD69 showed significant interactions with multiple cell types, and a key ligand receptor pair, CD99-PILRA, was identified, which may upregulate IL-6 and TNF-α upon T cell activation, potentially enhancing anti-tumor immunity or promoting immunosuppression [80].(Fig 7C). Notably, MIF-(CD74 + CXCR4) and MIF-(CD74 + CD44) were predicted as key ligand receptor pairs mediating macrophage T cell interactions across all three subtypes; MIF may induce metabolic reprogramming of tumor cells and generate an immunosuppressive microenvironment, and targeting MIF has been proposed to improve breast cancer immunotherapy [81](Fig 7D-F). Collectively, these analyses predicted complex macrophage T cell interaction networks across HER2 + , TNBC, and ER+ breast cancers and identified key ligand receptor pairs, including MIF-(CD74 + CXCR4), CD99-PILRA, and PTPRC-MRC1, involved in regulating immune cell infiltration, polarization, and function. This study also suggests a potential role of AnxA1 in contributing to an immunosuppressive microenvironment, potentially through inhibiting cytotoxic T cell responses and inducing Treg cells.

**Fig 7:**
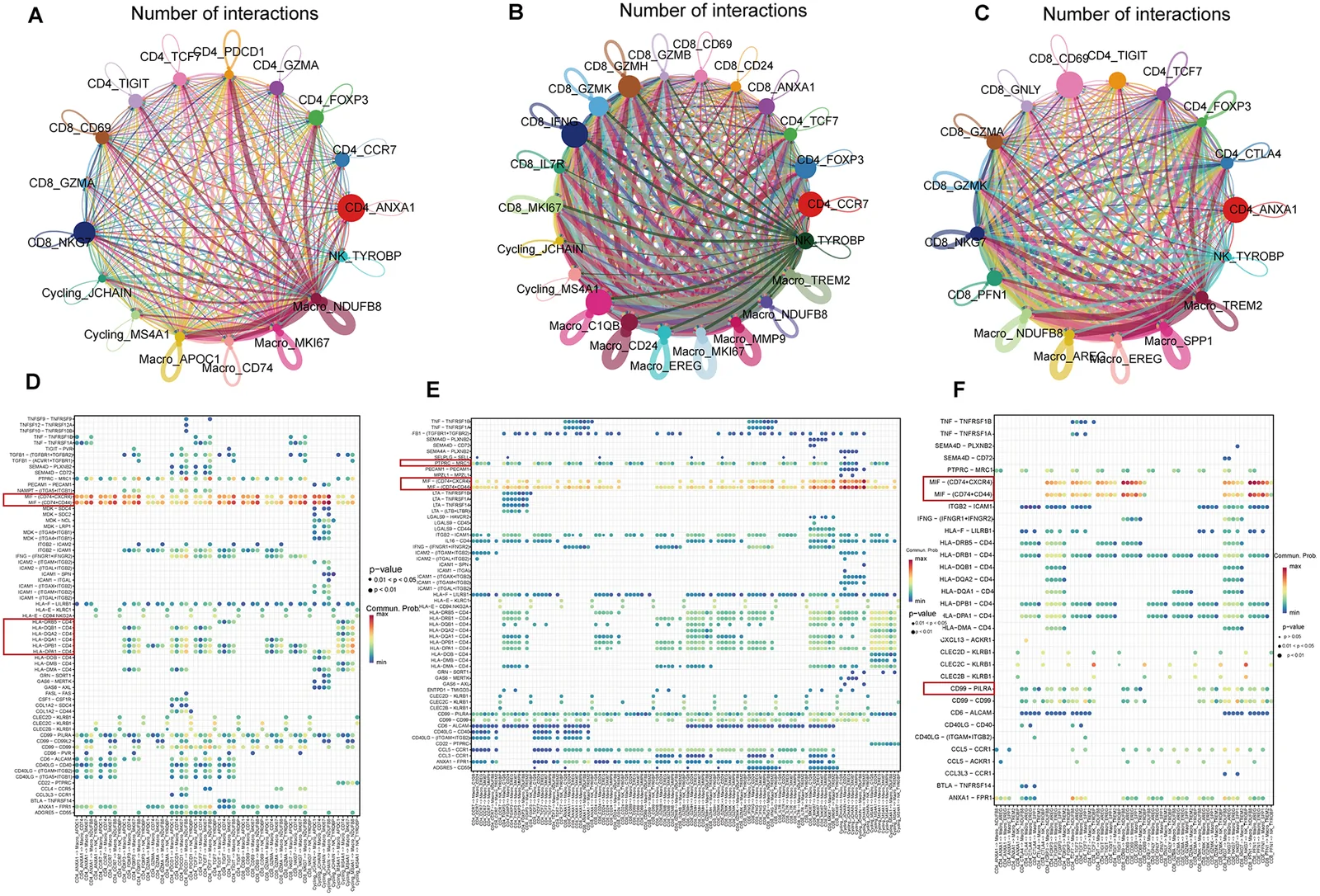
Macrophage and T-cell interactions in three subtypes of breast cancer. (A-C) The strength of the interactions between the three subtypes HER2 + ,TNBC,ER+ in that order, the size of the various coloured circles around the periphery indicates the number of cells, the larger the circle, the higher the number of cells. Cells emitting arrows express ligands, and cells pointed to by arrows express receptors. The more ligand-receptor pairs, the thicker the line; (D-F) Ligand-receptor interactions between macrophages and T cells of the three subtypes HER2 + ,TNBC,ER+ in that order.The parts marked with red borders represent the key ligand-receptor interaction pairs.

### 3.7. Constructing multicellular interaction atlas in the breast cancer immune microenvironment and analysing cross-subtype conserved networks and subtype-specific regulatory patterns

To further dissect the interactions among T cells, macrophages, and B cells across breast cancer subtypes, we employed CellChat to explore the crosstalk among these three immune cell types and constructed dynamic multicellular interaction networks in the breast cancer immune microenvironment (Fig 8). We identified eight cell subsets shared across the three subtypes: Memory_B, Plasma_B, Naive_B, Macro_NDUFB8, Macro_MKI67, CD4_FOXP3, CD8_CD69, and CD4_TCF7. These results revealed that these eight cell subsets constitute the core immune components common to HER2 + /TNBC/ER+ breast cancers, suggesting their involvement in universal immune regulatory programs and potential as general immunotherapeutic targets.Further analysis showed that Macro_NDUFB8 macrophages engaged in ligand-receptor interactions with multiple cell subsets across subtypes. Notably, the Macro_NDUFB8 macrophage subset engages in ligand-receptor interactions with multiple cell subsets across all three breast cancer subtypes. For instance, this subset can form sustained immune interactions with regulatory T cells (CD4_FOXP3) via MHC class II molecules (HLA-DRB5/DRB1/DQB1), and this interaction is conserved across the three subtypes, suggesting its potential involvement in a common pathway underlying immune suppression in breast cancer. These results indicate that this interaction may represent a potential conserved mechanism of immune escape in breast cancer, and Macro_NDUFB8 may exert immune regulatory hub-related functions. At a theoretical level, blocking the HLA-II-CD4 interaction could provide a novel approach to alleviating Treg-mediated immune suppression, and Macro_NDUFB8 holds promise as a potential target for combination therapy in breast cancer. However, its clinical application value requires further verification through in vitro and in vivo functional experiments. Subtype-specific shared subsets were also identified: CD4_CCR7 and Cycling_JCHAIN between HER2+ and TNBC; CD4_TIGIT, Macro_CD74, CD8_GZMA, CD8_NKG7, CD4_ANXA1, and NK_TYROBP between HER2+ and ER + ; and CD8_GZMK, Macro_EREG, and Macro_TREM2 between TNBC and ER + . Each subtype harbored unique cell subsets: HER2 + was characterized by CD4_PDCD1, Macro_APOC1, and CD4_GZMA, with Memory_B cells engaging in dynamic crosstalk with macrophages and T cells via ligand-receptor pairs such as PTPRC-CD55 and MIF-CD74 + CXCR4. TNBC-specific subsets included CD8_CD24, and CD8_GZMB, with Plasma_B cells forming interaction networks through MIF-(CD74 + CXCR4), ICAM1-SPN, and MHC class II-mediated interactions with CD4 + T cells. ER+ featured CD8_GNLY, CD8_PFN1, and CD4_CTLA4, with Naive_B cells interacting with T cells and macrophages via non-canonical pathways like ITGB2-ICAM1 and HLA-DRB1-CD4. Collectively, these findings delineate a pan-subtype core immune regulatory network centered on Macro_NDUFB8 and Tregs via MHC II-CD4 interactions, alongside subtype-specific networks, providing a framework for understanding immune escape and identifying potential therapeutic targets.

**Fig 8:**
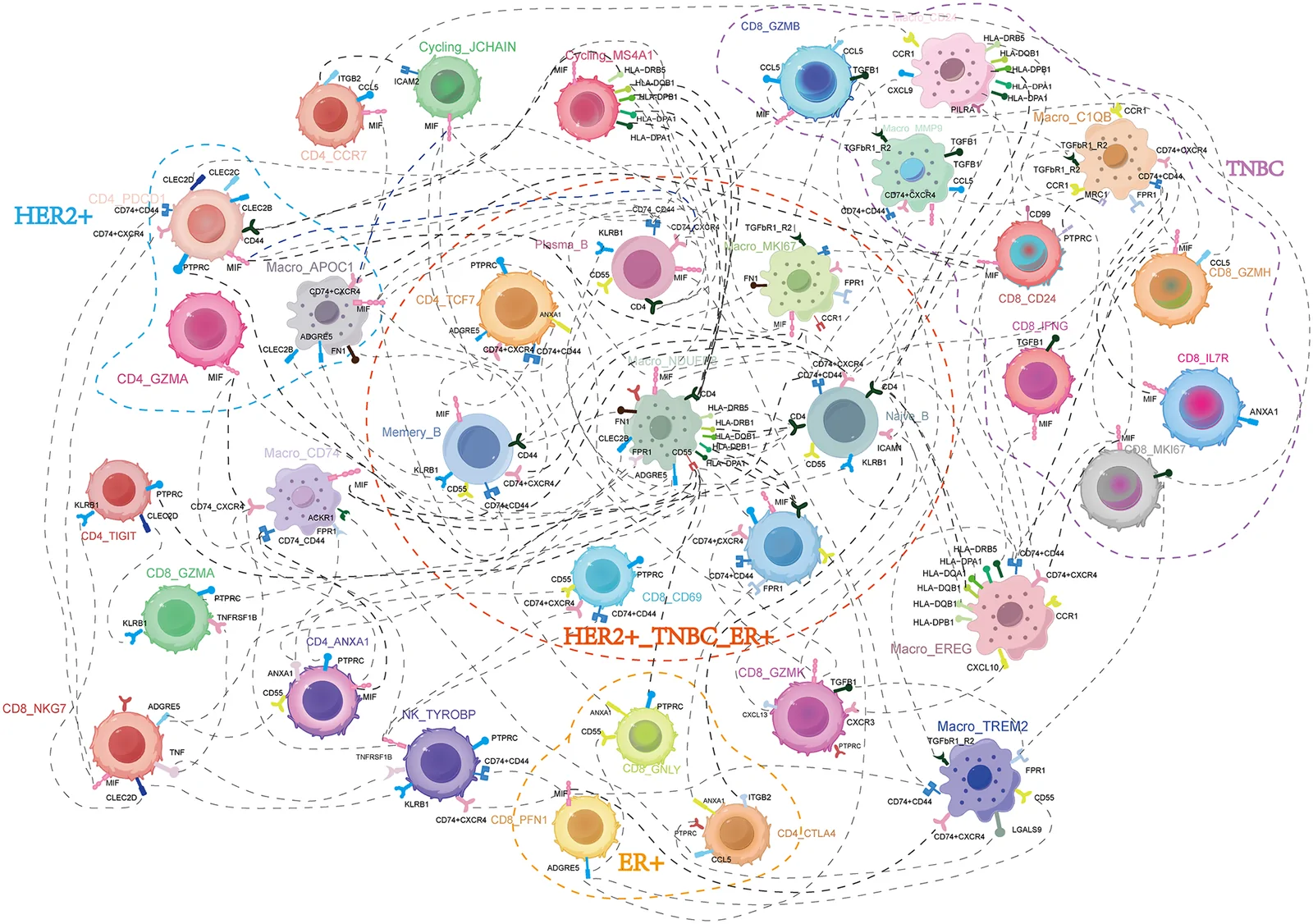
Construction of the multicellular interaction atlas in the breast cancer immune microenvironment. This figure shows the shared and subtype-specific cell-cell interaction networks among the three breast cancer subtypes (HER2 + , TNBC, and ER+). Cell subsets are labeled according to their distribution patterns across different subtypes: subsets shared by all three breast cancer subtypes are enclosed in orange dashed circles; HER2 + specific subsets are enclosed in blue dashed circles; TNBC specific subsets are enclosed in purple dashed circles; and ER+ specific subsets are enclosed in yellow dashed circles. Lines in the figure represent ligand-receptor interactions. This figure was created using Figdraw (http://www.figdraw.com).

## 4. Discussion

substantial heterogeneity across its molecular subtypes, yet the immune microenvironmental differences between HER2 + , TNBC, and ER+ tumors remain incompletely characterized. While recent studies have constructed single-cell and spatial atlases of human breast cancers [3] or investigated age-related alterations in the tumor microenvironment [82], these efforts have largely focused on cellular mapping or age-associated differences rather than directly comparing the functional states of immune cells across subtypes. In contrast, this study provides a systematic comparison of T cells, macrophages, and B cells across HER2 + , TNBC, and ER+ subtypes. By identifying subtype-specific transcriptional regulatory networks and conserved immune regulatory hubs, our findings offer new insights into the immune landscape of breast cancer.

Our analysis revealed that macrophages dominate the TME across the three breast cancer subtypes. Notably, macrophage subsets such as Macro_NDUFB8 exhibit significant subtype-dependent functional plasticity, manifested as exerting pro-tumor effects in HER2+ and TNBC, while exerting anti-tumor functions in ER + . This finding not only challenges the traditional M1/M2 macrophage dichotomy [83] and reveals the dynamic regulatory mechanism of the TME on immune cell function but also supplements the theoretical system of dendritic cell functional defects through the phenomenon of specific depletion of pDC in TNBC [84].

Furthermore, SCENIC analysis predicted that BCL11A may be linked to tumor proliferation in HER2+ and TNBC subtypes via its role in DNA repair [66]. Additionally, KLF6 and GATA6 might be involved in regulating the immune microenvironment in TNBC and ER⁺ tumors [69,70]. These candidate transcription factors offer potential clues for understanding the heterogeneity of immune cell regulation across breast cancer subtypes.

We further explored intercellular interaction networks and identified eight core immune cell subsets shared across subtypes. Macro_NDUFB8 macrophages may form an immune regulatory hub with Tregs via the MHC class II-CD4 pathway, suggesting a potential common immune escape mechanism. Additionally, subtype-specific interaction patterns were observed: Memory B cells in HER2 + might drive TME remodeling through PTPRC-CD55 and MIF pathways; Plasma cells in TNBC may dominate intercellular communication via MIF and MHC class II; and Naive B cells in ER+ could participate in immune regulation through non-canonical pathways such as ITGB2-ICAM1. Together, these findings suggest a potential role for B cells in subtype-specific immune regulation.

Several limitations of this study should be acknowledged. First, our findings regarding regulatory networks and ligand-receptor interactions are computational predictions and require experimental validation through functional assays, such as genetic perturbation or co-culture experiments. Second, single-cell transcriptomics lacks spatial context, which is essential for understanding cell-cell interactions in the tumor microenvironment. Third, potential sampling bias may exist due to the limited and uneven sample sizes across subtypes. Future studies with larger, more balanced cohorts are needed to validate our findings. Fourth, the survival analysis based on TCGA data provides only correlative evidence. Future studies should experimentally validate the candidate transcription factors identified in our analysis.

In conclusion, this study provides a systematic single-cell comparison of immune microenvironments across HER2 + , TNBC, and ER+ breast cancer subtypes. Our findings reveal subtype-specific immune cell compositions, conserved and subtype-specific immunoregulatory networks, and candidate transcription factors that may inform precision cancer therapy.

## Supporting information

S1 Fig Integrated heatmap of top 500 highly variable genes across shared cell types in breast cancer subtypes. Average expression of each gene in each cell type was calculated for each subtype (pseudobulking), and the top 500 highly variable genes shared across the three subtypes were selected. The matrix was z-score normalized across all subtypes, and columns are annotated by subtype and cell type. The heatmap highlights conserved transcriptional patterns of shared cell types while emphasizing subtype-specific differences, providing a focused and interpretable view of the most variable genes across subtypes.
(TIF)

S2 Fig Cell clustering and composition analysis of HER2 + , TNBC, and ER+ breast cancer subtypes. (A) tSNE plots of cell clusters in HER2 + , TNBC, and ER+ breast cancer cell lines. (B) Expression of classic marker genes in cell annotation. (C) Distribution preferences of cell types in HER2 + , TNBC, and ER+ breast cancer.
(TIF)

S3 Fig Analysis of the heterogeneity of tumor cell functions in HER2 + , TNBC, and ER+ breast cancer subtypes. (A) Differential analysis of tumor and non – tumor cells in HER2 + , TNBC, and ER+ breast cancer subtypes.(B) Analysis of up – and down – regulated functions of the differences between tumor and non – tumor cells in the three breast cancer subtypes.(C) Venn diagram showing the intersection of differential genes in the three breast cancer subtypes.
(TIF)

S4 Fig CytoTRACE2 analysis reveals distinct macrophage differentiation trajectories across HER2 + , TNBC, and ER+ breast cancer subtypes. (A) HER2 + , (B) TNBC, (C) ER + . Cells are colored by differentiation potency (blue: high, red: low). Trajectory lines indicate inferred differentiation paths. Terminal differentiation states (red) are subtype-dependent: pro-tumor in TNBC, anti-tumor in ER + , and mixed in HER2 + . Dual-function subsets localize to intermediate positions. Scale bar indicates potency score (0–1).
(TIF)

S5 Fig Expression of the NDUFB8 gene in macrophage subsets across three breast cancer subtypes. (A-C) Represent the expression of the NDUFB8 gene in macrophage subsets of HER2 + , TNBC, and ER + , respectively.
(TIF)

S6 Fig Integrated UMAP of macrophages from three breast cancer subtypes. Integrated UMAP showing macrophage populations from ER + , HER2 + , and TNBC tumors. Left: Cells colored by macrophage subclusters. Right: Cells colored by tumor molecular subtype. Macro_NDUFB8 cells from different tumor subtypes co-cluster and intermingle, indicating shared transcriptional identity of this subset across subtypes.
(TIF)

S7 Fig Patient-level pseudobulk expression of NDUFB8 across macrophage subpopulations. Violin plot of patient-level pseudobulk NDUFB8 expression in macrophage subpopulations. Each dot corresponds to one individual patient. NDUFB8 exhibits specific high expression in the Macro_NDUFB8 subset.
(TIF)

S8 Fig Gene signature scores of CD8 + T cell functional states across breast cancer subtypes. The heatmap displays enrichment scores of co-stimulatory molecules, immunosuppressive signature, dysfunctional signature, and cytotoxic signature across NK, CTL, Treg, TRM, naive, and Cycling T cell subsets. Color scale represents z-scored enrichment scores (blue: low enrichment; red: high enrichment).
(TIF)

S1 Table Key transcription factors identified based on macrophages, T cells, and B cells. Candidate transcription factors in macrophages, T cells, and B cells across breast cancer subtypes. This table lists subtype-specific transcription factors predicted by SCENIC analysis for each immune cell type in HER2 ⁺ , TNBC, and ER⁺ breast cancers. These TFs are presented as candidate regulators potentially contributing to immune cell heterogeneity
(XLSX)

S2 Table Functional enrichment analysis of the Macro_NDUFB8 subset across HER2 + , TNBC, and ER+ breast cancer subtypes. This table contains the functional enrichment analysis of the Macro_NDUFB8 subset in HER2 + , TNBC, and ER+ breast cancer.
(XLSX)

S3 Table Pathway enrichment analysis of differentially expressed genes in HER2 + , TNBC, and ER+breast cancer cells. This table shows the top enriched KEGG pathways for upregulated genes in HER2+ and TNBC tumor cells, and for downregulated genes in ER+ breast cancer cells.
(XLSX)

S4 Table Summary of immune cell subsets across HER2 + , TNBC, and ER+ breast cancer subtypes. This table lists major immune cell subsets, their subclusters, key marker genes, and subtype enrichment patterns. Subsets include T cells, macrophages, B cells, and other immune cells (dendritic cells, monocytes, cycling cells).
(XLSX)

## References

[1] Alshetaiwi H, et al. Defining the emergence of myeloid-derived suppressor cells in breast cancer using single-cell transcriptomics. Sci Immunol. 2020;5(44). 10.1126/sciimmunol.aay6017 PMC7219211 32086381

[2] Ishwar D, Premachandran S, Das S, Venkatakrishnan K, Tan B. Profiling breast tumor heterogeneity and identifying breast cancer subtypes through tumor-associated immune cell signatures and immuno nano sensors. Small. 2024;20(52):e2406475. doi: 10.1002/smll.202406475 39460487 PMC11673452

[3] Wu SZ, Al-Eryani G, Roden DL, Junankar S, Harvey K, Andersson A, et al. A single-cell and spatially resolved atlas of human breast cancers. Nat Genet. 2021;53(9):1334–47. doi: 10.1038/s41588-021-00911-1 34493872 PMC9044823

[4] Harris MA, Savas P, Virassamy B, O’Malley MMR, Kay J, Mueller SN, et al. Towards targeting the breast cancer immune microenvironment. Nat Rev Cancer. 2024;24(8):554–77. doi: 10.1038/s41568-024-00714-6 38969810

[5] Manohar SM, Shah P, Nair A. Flow cytometry: Principles, applications and recent advances. Bioanalysis. 2021;13(3):181–98. 10.4155/bio-2020-0267 33543666

[6] Liu CC, Steen CB, Newman AM. Computational approaches for characterizing the tumor immune microenvironment. Immunology. 2019;158(2):70–84. 10.1111/imm.13101 PMC6742767 31347163

[7] Fincham REA, et al. Editorial: Multiplex immunohistochemistry/immunofluorescence technique: The potential and promise for clinical application. Front Mol Biosci. 2022;9:831383. 10.3389/fmolb.2022.831383 PMC8914508 35281257

[8] Kulkarni A, Anderson AG, Merullo DP, Konopka G. Beyond bulk: A review of single cell transcriptomics methodologies and applications. Curr Opin Biotechnol. 2019;58:129–36. doi: 10.1016/j.copbio.2019.03.001 30978643 PMC6710112

[9] Chung W, Eum HH, Lee H-O, Lee K-M, Lee H-B, Kim K-T, et al. Single-cell RNA-seq enables comprehensive tumour and immune cell profiling in primary breast cancer. Nat Commun. 2017;8:15081. doi: 10.1038/ncomms15081 28474673 PMC5424158

[10] Azizi E, Carr AJ, Plitas G, Cornish AE, Konopacki C, Prabhakaran S, et al. Single-cell map of diverse immune phenotypes in the breast tumor microenvironment. Cell. 2018;174(5):1293-1308.e36. doi: 10.1016/j.cell.2018.05.060 29961579 PMC6348010

[11] Zhang N, Bevan MJ. CD8(+) T cells: Foot soldiers of the immune system. Immunity. 2011;35(2):161–8. doi: 10.1016/j.immuni.2011.07.010 21867926 PMC3303224

[12] Chitnis T. The role of CD4 T cells in the pathogenesis of multiple sclerosis. Int Rev Neurobiol. 2007;79:43–72. doi: 10.1016/S0074-7742(07)79003-7 17531837 PMC7112308

[13] Downs-Canner SM, Meier J, Vincent BG, Serody JS. B cell function in the tumor microenvironment. Annu Rev Immunol. 2022;40:169–93. doi: 10.1146/annurev-immunol-101220-015603 35044794

[14] Mantovani A, Allavena P, Marchesi F, Garlanda C. Macrophages as tools and targets in cancer therapy. Nat Rev Drug Discov. 2022;21(11):799–820. doi: 10.1038/s41573-022-00520-5 35974096 PMC9380983

[15] Zhang Y, Zhong F, Liu L. Single-cell transcriptional atlas of tumor-associated macrophages in breast cancer. Breast Cancer Res. 2024;26(1):129. doi: 10.1186/s13058-024-01887-6 39232806 PMC11373130

[16] Honkanen TJ, Tikkanen A, Karihtala P, Mäkinen M, Väyrynen JP, Koivunen JP. Prognostic and predictive role of tumour-associated macrophages in HER2 positive breast cancer. Sci Rep. 2019;9(1):10961. doi: 10.1038/s41598-019-47375-2 31358801 PMC6662906

[17] Peralta RM, Xie B, Lontos K, Nieves-Rosado H, Spahr K, Joshi S, et al. Dysfunction of exhausted T cells is enforced by MCT11-mediated lactate metabolism. Nat Immunol. 2024;25(12):2297–307. doi: 10.1038/s41590-024-01999-3 39516648 PMC11588660

[18] Guo S, Liu X, Zhang J, Huang Z, Ye P, Shi J, et al. Integrated analysis of single-cell RNA-seq and bulk RNA-seq unravels T cell-related prognostic risk model and tumor immune microenvironment modulation in triple-negative breast cancer. Comput Biol Med. 2023;161:107066. doi: 10.1016/j.compbiomed.2023.107066 37263064

[19] Lee S, Cho Y, Li Y, Li R, Lau AW, Laird MS, et al. Cancer-cell derived S100A11 promotes macrophage recruitment in ER+ breast cancer. Oncoimmunology. 2024;13(1):2429186. doi: 10.1080/2162402X.2024.2429186 39587886 PMC11601052

[20] Wang Q, Sun K, Liu R, Song Y, Lv Y, Bi P, et al. Single-cell transcriptome sequencing of B-cell heterogeneity and tertiary lymphoid structure predicts breast cancer prognosis and neoadjuvant therapy efficacy. Clin Transl Med. 2023;13(8):e1346. doi: 10.1002/ctm2.1346 37525587 PMC10390819

[21] Papanicolaou M, Parker AL, Yam M, Filipe EC, Wu SZ, Chitty JL, et al. Temporal profiling of the breast tumour microenvironment reveals collagen XII as a driver of metastasis. Nat Commun. 2022;13(1):4587. doi: 10.1038/s41467-022-32255-7 35933466 PMC9357007

[22] Malighetti F, Villa M, Villa AM, Pelucchi S, Aroldi A, Cortinovis DL, et al. Prognostic biomarkers in breast cancer via multi-omics clustering analysis. Int J Mol Sci. 2025;26(5):1943. doi: 10.3390/ijms26051943 40076569 PMC11900291

[23] Jenkins EO, Deal AM, Anders CK, Prat A, Perou CM, Carey LA, et al. Age-specific changes in intrinsic breast cancer subtypes: A focus on older women. Oncologist. 2014;19(10):1076–83. doi: 10.1634/theoncologist.2014-0184 25142841 PMC4200998

[24] Ogata H, et al. KEGG: Kyoto encyclopedia of genes and genomes. Nucleic Acids Res. 1999;27(1):29–34. 10.1093/nar/27.1.29 PMC148090 9847135

[25] Kanehisa M. Toward understanding the origin and evolution of cellular organisms. Protein Sci. 2019;28(11):1947–51. doi: 10.1002/pro.3715 31441146 PMC6798127

[26] Kanehisa M, Furumichi M, Sato Y, Kawashima M, Ishiguro-Watanabe M. KEGG for taxonomy-based analysis of pathways and genomes. Nucleic Acids Res. 2023;51(D1):D587–92. doi: 10.1093/nar/gkac963 36300620 PMC9825424

[27] Hanahan D, Weinberg RA. Hallmarks of cancer: The next generation. Cell. 2011;144(5):646–74. doi: 10.1016/j.cell.2011.02.013 21376230

[28] Houseley J, Tollervey D. The many pathways of RNA degradation. Cell. 2009;136(4):763–76. doi: 10.1016/j.cell.2009.01.019 19239894

[29] Malumbres M, Barbacid M. Cell cycle, CDKs and cancer: A changing paradigm. Nat Rev Cancer. 2009;9(3):153–66. doi: 10.1038/nrc2602 19238148

[30] Hajizadeh F, Aghebati Maleki L, Alexander M, Mikhailova MV, Masjedi A, Ahmadpour M, et al. Tumor-associated neutrophils as new players in immunosuppressive process of the tumor microenvironment in breast cancer. Life Sci. 2021;264:118699. doi: 10.1016/j.lfs.2020.118699 33137368

[31] Nian Z, Dou Y, Shen Y, Liu J, Du X, Jiang Y, et al. Interleukin-34-orchestrated tumor-associated macrophage reprogramming is required for tumor immune escape driven by p53 inactivation. Immunity. 2024;57(10):2344-2361.e7. doi: 10.1016/j.immuni.2024.08.015 39321806

[32] Brett JO, Spring LM, Bardia A, Wander SA. ESR1 mutation as an emerging clinical biomarker in metastatic hormone receptor-positive breast cancer. Breast Cancer Res. 2021;23(1):85. doi: 10.1186/s13058-021-01462-3 34392831 PMC8365900

[33] Zhao C, Cai X, Wang Y, Wang D, Wang T, Gong H, et al. NAT1 promotes osteolytic metastasis in luminal breast cancer by regulating the bone metastatic niche via NF-κB/IL-1B signaling pathway. Am J Cancer Res. 2020;10(8):2464–79. 32905535 PMC7471372

[34] Salman Yaylaz B, Sırma Ekmekci S, Çınar S, Deniz G, Abacı N. DHRS2 is a potential marker of breast cancer metastasis. Gene Reports. 2021;25:101302. doi: 10.1016/j.genrep.2021.101302

[35] Niu X, Zhan Y, Zhang S, Liu Z, Qu C. Research progress of STC2 in breast cancer. Biophys Rep. 2021;7(3):185–92. doi: 10.52601/bpr.2021.210002 37287492 PMC10244795

[36] Jansen MPHM, Sas L, Sieuwerts AM, Van Cauwenberghe C, Ramirez-Ardila D, Look M, et al. Decreased expression of ABAT and STC2 hallmarks ER-positive inflammatory breast cancer and endocrine therapy resistance in advanced disease. Mol Oncol. 2015;9(6):1218–33. doi: 10.1016/j.molonc.2015.02.006 25771305 PMC5528763

[37] Tokumaru Y, Oshi M, Katsuta E, Yan L, Satyananda V, Matsuhashi N, et al. KRAS signaling enriched triple negative breast cancer is associated with favorable tumor immune microenvironment and better survival. Am J Cancer Res. 2020;10(3):897–907. 32266098 PMC7136911

[38] Tsoi H, Man EPS, Chau KM, Khoo U-S. Targeting the IL-6/STAT3 signalling cascade to reverse tamoxifen resistance in estrogen receptor positive breast cancer. Cancers (Basel). 2021;13(7):1511. doi: 10.3390/cancers13071511 33806019 PMC8036560

[39] Rodriguez-Barrueco R, Yu J, Saucedo-Cuevas LP, Olivan M, Llobet-Navas D, Putcha P, et al. Inhibition of the autocrine IL-6-JAK2-STAT3-calprotectin axis as targeted therapy for HR-/HER2+ breast cancers. Genes Dev. 2015;29(15):1631–48. doi: 10.1101/gad.262642.115 26227964 PMC4536311

[40] Chang R, Song L, Xu Y, Wu Y, Dai C, Wang X, et al. Loss of Wwox drives metastasis in triple-negative breast cancer by JAK2/STAT3 axis. Nat Commun. 2018;9(1):3486. doi: 10.1038/s41467-018-05852-8 30154439 PMC6113304

[41] Huang B, Lang X, Li X. The role of IL-6/JAK2/STAT3 signaling pathway in cancers. Front Oncol. 2022;12:1023177. doi: 10.3389/fonc.2022.1023177 36591515 PMC9800921

[42] Zimmerli D, Brambillasca CS, Talens F, Bhin J, Linstra R, Romanens L, et al. MYC promotes immune-suppression in triple-negative breast cancer via inhibition of interferon signaling. Nat Commun. 2022;13(1):6579. doi: 10.1038/s41467-022-34000-6 36323660 PMC9630413

[43] Broz ML, Binnewies M, Boldajipour B, Nelson AE, Pollack JL, Erle DJ, et al. Dissecting the tumor myeloid compartment reveals rare activating antigen-presenting cells critical for T cell immunity. Cancer Cell. 2014;26(5):638–52. doi: 10.1016/j.ccell.2014.09.007 25446897 PMC4254577

[44] Allen BM, Hiam KJ, Burnett CE, Venida A, DeBarge R, Tenvooren I, et al. Systemic dysfunction and plasticity of the immune macroenvironment in cancer models. Nat Med. 2020;26(7):1125–34. doi: 10.1038/s41591-020-0892-6 32451499 PMC7384250

[45] Gordon SR, Maute RL, Dulken BW, Hutter G, George BM, McCracken MN, et al. PD-1 expression by tumour-associated macrophages inhibits phagocytosis and tumour immunity. Nature. 2017;545(7655):495–9. doi: 10.1038/nature22396 28514441 PMC5931375

[46] Ruffell B, Coussens LM. Macrophages and therapeutic resistance in cancer. Cancer Cell. 2015;27(4):462–72. doi: 10.1016/j.ccell.2015.02.015 25858805 PMC4400235

[47] Ferrara N, Gerber H-P, LeCouter J. The biology of VEGF and its receptors. Nat Med. 2003;9(6):669–76. doi: 10.1038/nm0603-669 12778165

[48] Neo SY, Jing X, Tong L, Tong D, Gao J, Chen Z, et al. Tumor MHC class I expression alters cancer-associated myelopoiesis driven by host NK cells. J Immunother Cancer. 2022;10(10):e005308. doi: 10.1136/jitc-2022-005308 36283735 PMC9608525

[49] Roche PA, Furuta K. The ins and outs of MHC class II-mediated antigen processing and presentation. Nat Rev Immunol. 2015;15(4):203–16. doi: 10.1038/nri3818 25720354 PMC6314495

[50] Greenwald RJ, Freeman GJ, Sharpe AH. The B7 family revisited. Annu Rev Immunol. 2005;23:515–48. 10.1146/annurev.immunol.23.021704.115611 15771580

[51] Dinarello CA. Interleukin-1 in the pathogenesis and treatment of inflammatory diseases. Blood. 2011;117(14):3720–32. doi: 10.1182/blood-2010-07-273417 21304099 PMC3083294

[52] Mantovani A, Locati M. Tumor-associated macrophages as a paradigm of macrophage plasticity, diversity, and polarization: Lessons and open questions. Arterioscler Thromb Vasc Biol. 2013;33(7):1478–83. doi: 10.1161/ATVBAHA.113.300168 23766387

[53] Veglia F, Sanseviero E, Gabrilovich DI. Myeloid-derived suppressor cells in the era of increasing myeloid cell diversity. Nat Rev Immunol. 2021;21(8):485–98. doi: 10.1038/s41577-020-00490-y 33526920 PMC7849958

[54] Batlle E, Massagué J. Transforming growth factor-β signaling in immunity and cancer. Immunity. 2019;50(4):924–40. doi: 10.1016/j.immuni.2019.03.024 30995507 PMC7507121

[55] Etzerodt A, Moestrup SK. CD163 and inflammation: Biological, diagnostic, and therapeutic aspects. Antioxid Redox Signal. 2013;18(17):2352–63. doi: 10.1089/ars.2012.4834 22900885 PMC3638564

[56] Hu H, Cheng R, Wang Y, Wang X, Wu J, Kong Y, et al. Oncogenic KRAS signaling drives evasion of innate immune surveillance in lung adenocarcinoma by activating CD47. J Clin Invest. 2023;133(2):e153470. doi: 10.1172/JCI153470 36413402 PMC9843062

[57] Garcia SAB, Araújo M, Freitas R. Dataset of HOXB7, HOXB8 and HOXB9 expression profiles in cell lines representative of the breast cancer molecular subtypes Luminal a (MCF7), Luminal b (BT474), HER2+ (SKBR3) and triple-negative (MDA231, MDA468), compared to a model of normal cells (MCF10A). Data Brief. 2020;30:105572. doi: 10.1016/j.dib.2020.105572 32346580 PMC7182708

[58] Andrechek ER. HER2/Neu tumorigenesis and metastasis is regulated by E2F activator transcription factors. Oncogene. 2015;34(2):217–25. doi: 10.1038/onc.2013.540 24362522 PMC4067469

[59] Lu Y, Su F, Yang H, Xiao Y, Zhang X, Su H, et al. E2F1 transcriptionally regulates CCNA2 expression to promote triple negative breast cancer tumorigenicity. Cancer Biomark. 2022;33(1):57–70. doi: 10.3233/CBM-210149 34366326 PMC12364124

[60] Kim Y, Kim H-S, Do S-I. Low nuclear zinc finger and BTB domain containing 7A expression is an independent prognostic factor for recurrence-free survival in invasive ductal carcinoma of the breast. Int J Clin Exp Pathol. 2018;11(4):2193–200. 31938331 PMC6958227

[61] Mao A, Chen M, Qin Q, Liang Z, Jiang W, Yang W, et al. ZBTB7A promotes migration, invasion and metastasis of human breast cancer cells through NF-κB-induced epithelial-mesenchymal transition in vitro and in vivo. J Biochem. 2019;166(6):485–93. doi: 10.1093/jb/mvz062 31385585

[62] Mazloumi Z, Rafat A, Dizaji Asl K, Nozad Charoudeh H. A combination of telomerase inhibition and NK cell therapy increased breast cancer cell line apoptosis. Biochem Biophys Res Commun. 2023;640:50–5. doi: 10.1016/j.bbrc.2022.11.090 36502631

[63] Somasundaram V, Ridnour LA, Cheng RY, Walke AJ, Kedei N, Bhattacharyya DD, et al. Systemic Nos2 Depletion and Cox inhibition limits TNBC disease progression and alters lymphoid cell spatial orientation and density. Redox Biol. 2022;58:102529. doi: 10.1016/j.redox.2022.102529 36375380 PMC9661390

[64] Kwa MJ, Adams S. Checkpoint inhibitors in triple-negative breast cancer (TNBC): Where to go from here. Cancer. 2018;124(10):2086–103. doi: 10.1002/cncr.31272 29424936

[65] Kim I, Sanchez K, McArthur HL, Page D. Immunotherapy in triple-negative breast cancer: Present and future. Curr Breast Cancer Rep. 2019;11(4):259–71. doi: 10.1007/s12609-019-00345-z

[66] Vickridge E, Faraco CCF, Tehrani PS, Ramdzan ZM, Rahimian H, Leduy L, et al. The DNA repair function of BCL11A suppresses senescence and promotes continued proliferation of triple-negative breast cancer cells. NAR Cancer. 2022;4(4):zcac028. doi: 10.1093/narcan/zcac028 36186110 PMC9516615

[67] Lanigan F, Gremel G, Hughes R, Brennan DJ, Martin F, Jirström K, et al. Homeobox transcription factor muscle segment homeobox 2 (Msx2) correlates with good prognosis in breast cancer patients and induces apoptosis in vitro. Breast Cancer Res. 2010;12(4):R59. doi: 10.1186/bcr2621 20682066 PMC2949651

[68] Wei Y, Wang X, Zhang Z, Zhao C, Chang Y, Bian Z, et al. Impact of NR5A2 and RYR2 3’UTR polymorphisms on the risk of breast cancer in a Chinese Han population. Breast Cancer Res Treat. 2020;183(1):1–8. doi: 10.1007/s10549-020-05736-w 32572717

[69] Xia B, Hou L, Kang H, Chang W, Liu Y, Zhang Y, et al. NR2F2 plays a major role in insulin-induced epithelial-mesenchymal transition in breast cancer cells. BMC Cancer. 2020;20(1):626. doi: 10.1186/s12885-020-07107-6 32631390 PMC7336611

[70] Liu J, Du T, Yuan Y, He Y, Tan Z, Liu Z. KLF6 inhibits estrogen receptor-mediated cell growth in breast cancer via a c-Src-mediated pathway. Mol Cell Biochem. 2010;335(1–2):29–35. doi: 10.1007/s11010-009-0237-8 19707857

[71] Song Y, Tian T, Fu X, Wang W, Li S, Shi T, et al. GATA6 is overexpressed in breast cancer and promotes breast cancer cell epithelial-mesenchymal transition by upregulating slug expression. Exp Mol Pathol. 2015;99(3):617–27. doi: 10.1016/j.yexmp.2015.10.005 26505174

[72] Saha SK, Islam SMR, Saha T, Nishat A, Biswas PK, Gil M, et al. Prognostic role of EGR1 in breast cancer: A systematic review. BMB Rep. 2021;54(10):497–504. doi: 10.5483/BMBRep.2021.54.10.087 34488929 PMC8560464

[73] Nutt SL, Hodgkin PD, Tarlinton DM, Corcoran LM. The generation of antibody-secreting plasma cells. Nat Rev Immunol. 2015;15(3):160–71. doi: 10.1038/nri3795 25698678

[74] Weisel F, Shlomchik M. Memory B cells of mice and humans. Annu Rev Immunol. 2017;35:255–84. doi: 10.1146/annurev-immunol-041015-055531 28142324

[75] He Z-Y, Wu S-G, Peng F, Zhang Q, Luo Y, Chen M, et al. Up-Regulation of RFC3 promotes triple negative breast cancer metastasis and is associated with poor prognosis via EMT. Transl Oncol. 2017;10(1):1–9. doi: 10.1016/j.tranon.2016.10.004 27888707 PMC5123039

[76] Liu H, Zhang J, Zhao Y, Fan Z, Yang Y, Mao Y, et al. CD93 regulates breast cancer growth and vasculogenic mimicry through the PI3K/AKT/SP2 signaling pathway activated by integrin β1. J Biochem Mol Toxicol. 2024;38(4):e23688. doi: 10.1002/jbt.23688 38511888

[77] Kruse B, Buzzai AC, Shridhar N, Braun AD, Gellert S, Knauth K, et al. CD4+ T cell-induced inflammatory cell death controls immune-evasive tumours. Nature. 2023;618(7967):1033–40. doi: 10.1038/s41586-023-06199-x 37316667 PMC10307640

[78] Li P, Wang W, Wang S, Cao G, Pan T, Huang Y, et al. PTPRC promoted CD8+ T cell mediated tumor immunity and drug sensitivity in breast cancer: Based on pan-cancer analysis and artificial intelligence modeling of immunogenic cell death-based drug sensitivity stratification. Front Immunol. 2023;14:1145481. doi: 10.3389/fimmu.2023.1145481 37388747 PMC10302730

[79] Squadrito ML, Pucci F, Magri L, Moi D, Gilfillan GD, Ranghetti A, et al. miR-511-3p modulates genetic programs of tumor-associated macrophages. Cell Rep. 2012;1(2):141–54. doi: 10.1016/j.celrep.2011.12.005 22832163

[80] Takheaw N, Earwong P, Laopajon W, Pata S, Kasinrerk W. Interaction of CD99 and its ligand upregulates IL-6 and TNF-α upon T cell activation. PLoS One. 2019;14(5):e0217393. doi: 10.1371/journal.pone.0217393 31120992 PMC6532917

[81] Yan L, Wu M, Wang T, Yuan H, Zhang X, Zhang H, et al. Breast cancer stem cells secrete MIF to mediate tumor metabolic reprogramming that drives immune evasion. Cancer Res. 2024;84(8):1270–85. doi: 10.1158/0008-5472.CAN-23-2390 38335272

[82] Parsons A, Sauras Colón E, Manjunath M, Zhang H, Chen J, Spasic M, et al. Cell populations in human breast cancers are molecularly and biologically distinct with age. Nat Aging. 2025;5(12):2546–63. doi: 10.1038/s43587-025-00984-1 41188599 PMC12705435

[83] Liu J, Geng X, Hou J, Wu G. New insights into M1/M2 macrophages: Key modulators in cancer progression. Cancer Cell Int. 2021;21(1):389. doi: 10.1186/s12935-021-02089-2 34289846 PMC8296555

[84] Michea P, Noël F, Zakine E, Czerwinska U, Sirven P, Abouzid O, et al. Adjustment of dendritic cells to the breast-cancer microenvironment is subset specific. Nat Immunol. 2018;19(8):885–97. doi: 10.1038/s41590-018-0145-8 30013147

